# A Cyclic Disulfide Diastereomer From Bioactive Fraction of *Bruguiera gymnorhiza* Shows Anti–*Pseudomonas aeruginosa* Activity

**DOI:** 10.3389/fphar.2022.890790

**Published:** 2022-06-02

**Authors:** Nilesh Lakshman Dahibhate, Sanjeev K. Shukla, Kundan Kumar

**Affiliations:** ^1^ Department of Biological Sciences, Birla Institute of Technology & Science Pilani, K. K. Birla Goa Campus, Goa, India; ^2^ Sophisticated Analytical Instrument Facility, CSIR-Central Drug Research Institute, Lucknow, India

**Keywords:** *Bruguiera gymnorhiza*, Brugierol, Isobrugierol, *Pseudomonas aeruginosa*, anti-quorum sensing, biofilm, cell viability

## Abstract

*Pseudomonas aeruginosa* is an opportunistic pathogen that commonly causes hospital-acquired infection and is of great concern in immunocompromised patients. The quorum sensing (QS) mechanism of *P. aeruginosa* is well studied and known to be responsible for pathogenicity and virulence. The QS inhibitor derived from the natural product can be an important therapeutic agent for pathogen control. The present study reports the role of *Bruguiera gymnorhiza* purified fraction (BG138) in inhibiting virulence factor production, biofilm formation, quorum sensing molecules, and expression of QS-related genes of *P. aeruginosa*. Structural characterization of BG138 by high resolution mass spectrometry, Fourier transform infrared spectroscopy, 1D (^1^H and ^13^C NMR) and 2D NMR reveals that the fraction is a mixture of already known cyclic disulfide diastereomer, namely, brugierol and isobrugierol. The minimum inhibitory concentration (MIC) of BG138 against *P. aeruginosa* was 32 μg/ml. Biofilm formation was significantly reduced at sub-MIC concentrations of BG138. Scanning electron microscopy analysis reports the concentration-dependent biofilm inhibition and morphological changes of *P. aeruginosa*. Flow cytometry–based cell viability assay showed that *P. aeruginosa* cells exhibit increased propidium iodide uptake on treatment with 32 and 64 μg/ml of BG138. At sub-MIC concentrations, BG138 exhibited significant inhibition of virulence factors and reduced swimming and swarming motility of *P. aeruginosa*. Furthermore, the effect of BG138 on the expression of QS-related genes was investigated by qRT-PCR. Taken together, our study reports the isolation and structural characterization of bioactive fraction BG138 from *B. gymnorhiza* and its anti-biofilm, anti-virulence, anti-quorum sensing, and cell-damaging activities against *P. aeruginosa*.

## 1 Introduction

Pathogenic and environmental bacteria can establish a stable biofilm under various conditions. Biofilm formation by pathogenic organisms is considered an advantage over other free-living or non–biofilm forming bacterial cells. It helps the organisms build resistance toward different antibiotics (Muhammad et al., 2020). Biofilms formed on food surfaces or medical equipment can result in possible foodborne diseases or infections (Quintieri et al., 2019). There are numerous pathogenic bacterial species capable of forming a biofilm, such as *Salmonella*, *Bacillus*, *Campylobacter*, and *Pseudomonas* species. *Pseudomonas aeruginosa* is a major cause of nosocomial infections and a leading pathogen among patients with cystic fibrosis and individuals with compromised immune defense (Moradali et al., 2017; Stefani et al., 2017). *P. aeruginosa* infections are complicated due to its intrinsic or acquired resistance to various antibiotics including carbapenems, aminoglycosides, and cephalosporins (Pang et al., 2019). The pathogenicity of *P. aeruginosa* is mainly due to the production of various virulence factors, including pyocyanin, elastase, protease, rhamnolipids, and alginate and, most importantly, the ability to form biofilm, which leads to development of antimicrobial resistance (Hu et al., 2017; Azam and Khan, 2019).

Biofilm formation and expression of virulence factors is regulated by quorum sensing (QS) (Pena et al., 2019; Kostylev et al., 2019). In many pathogenic bacteria, the QS system coordinates the bacterial motility, biofilm, and exotoxin production and plays a significant role in bacterial pathogenesis and food spoilage (Skandamis et al., 2012). In *P. aeruginosa*, synthesis of two acyl homoserine lactone (AHL) signaling molecules such as C4-HSL (*N*-butanoyl-L-homoserine lactone) and 3-oxo-C12-HSL (*N*-(3-oxododecanoyl)-L-homoserine lactone) are modulated by two sets of QS systems, such as lasI-lasR and rhlI-rhlR (Zhou et al., 2018). Bacterial strains with mutation in key genes of Las and Rhl-QS form an undifferentiated and unusual biofilm (Lin et al., 2018; Fong et al., 2019). Various synthetic AHL analogs (Kitamura et al., 2016; Soukarieh et al., 2018) and natural compounds such as norspermidine (Qu et al., 2016), hordenine (Zhou et al., 2018), and emodic acid (Ahmad et al., 2019) showed anti-QS, anti-biofilm, and anti-virulence properties. Therefore, natural products can be screened in search of novel active metabolites having anti-bacterial, anti-biofilm, and anti-QS properties (Asfour, 2018).

Mangrove ecosystems are widespread across the tropical and subtropical regions of the world in extreme climatic and environmental conditions such as high salinity and low oxygen (Saddhe et al., 2016). They are endowed with structurally diverse and biologically important secondary metabolites to sustain under biotic and abiotic stress (Dahibhate et al., 2019; Dahibhate et al., 2020). The mangrove species *Bruguiera gymnorhiza* belongs to a Rhizophoraceae family and mainly grows in the intermediate estuarine regions (Sadhhe et al., 2017). The leaves and roots of *B. gymnorhiza* are traditionally used for various therapeutic applications including astringent, diarrhea, malaria, and burns (Dahibhate et al., 2021). Previous studies on *B. gymnorhiza* have shown the presence of various secondary metabolites, including polyphenols, fatty acids, tannins, saponins, steroids, and terpenes (Nebula et al., 2013; Dahibhate et al., 2022). Besides, various therapeutic activities such as anti-oxidant, anticancer, anti-microbial, and anti-diabetic activities were reported (Patra et al., 2011; Mahmud et al., 2017). Furthermore, the key metabolites identified from *B. gymnorhiza* are gymnorrhizol, brugierol, isobrugierol, brugunin a, and bruguiesulfurol. Some of these isolated metabolites have anticancer (HepG2 cell line) and anti-bacterial activity against *Bacillus subtilis* and inhibitory activity against protein tyrosine phosphatase 1B (Homhual et al., 2006; Huang et al., 2009; Nebula et al., 2013). In addition, few authors reported crude extract of mangrove leaves having anti-*P. aeruginosa* (Nebula et al., 2013; Dahibhate et al., 2020), anti-quorum sensing, and anti-virulence activity (Annapoorani et al., 2013).

Even though mangrove species have been investigated for different therapeutic properties, only few mangrove species were reported to have antibacterial activities. However, to the best of our knowledge, none of the active metabolites isolated from *B. gymnorhiza* was demonstrated to inhibit biofilm formation, QS mechanism, and cell damage in *P. aeruginosa*. Here, we explored the effects of a bioactive fraction of *B. gymnorhiza*, henceforth referred to as BG138, which is a mixture of brugierol/isobrugierol on *P. aeruginosa* biofilm formation, virulence factor, auto-inducer signaling molecules, and QS-related gene expression.

## 2 Materials and Methods

### 2.1 Chemicals and Solvents

The primers were purchased from Eurofins Genomics, India. Hexane, ethyl acetate, chloroform, methanol, acetonitrile (Finar Chemicals, India), C4-HSL, 3-oxo-C12-HSL, formic acid, trichloroacetic acid, trifluroacetic acid, orcinol, azocasein, and carbazol were purchased (Sigma-Aldrich, St. Louis, MI, United States).

### 2.2 Collection and Preparation of *B. gymnorhiza* Extracts

*B. gymnorhiza* (L) Lam. leaves were collected from Achara region with the geographical latitude of 16°11'54"N and longitude of 73°26'42"E, Maharashtra, India, and authenticated at the taxonomy division, Shivaji University Kolhapur, Maharashtra, India (Voucher number: NLD-1).

The freshly collected leaves were dried (30°C) for 15 days and stored in airtight glass jars at 4°C. Dried powder of leaves (2.5 kg) was extracted with hexane, ethyl acetate, and methanol for 12 h. The solvent extracts obtained were evaporated using a rotary evaporator below 50°C and stored at 4°C.

### 2.3 Activity Guided Isolation of Bioactive Compound

**FIGURE 1:**
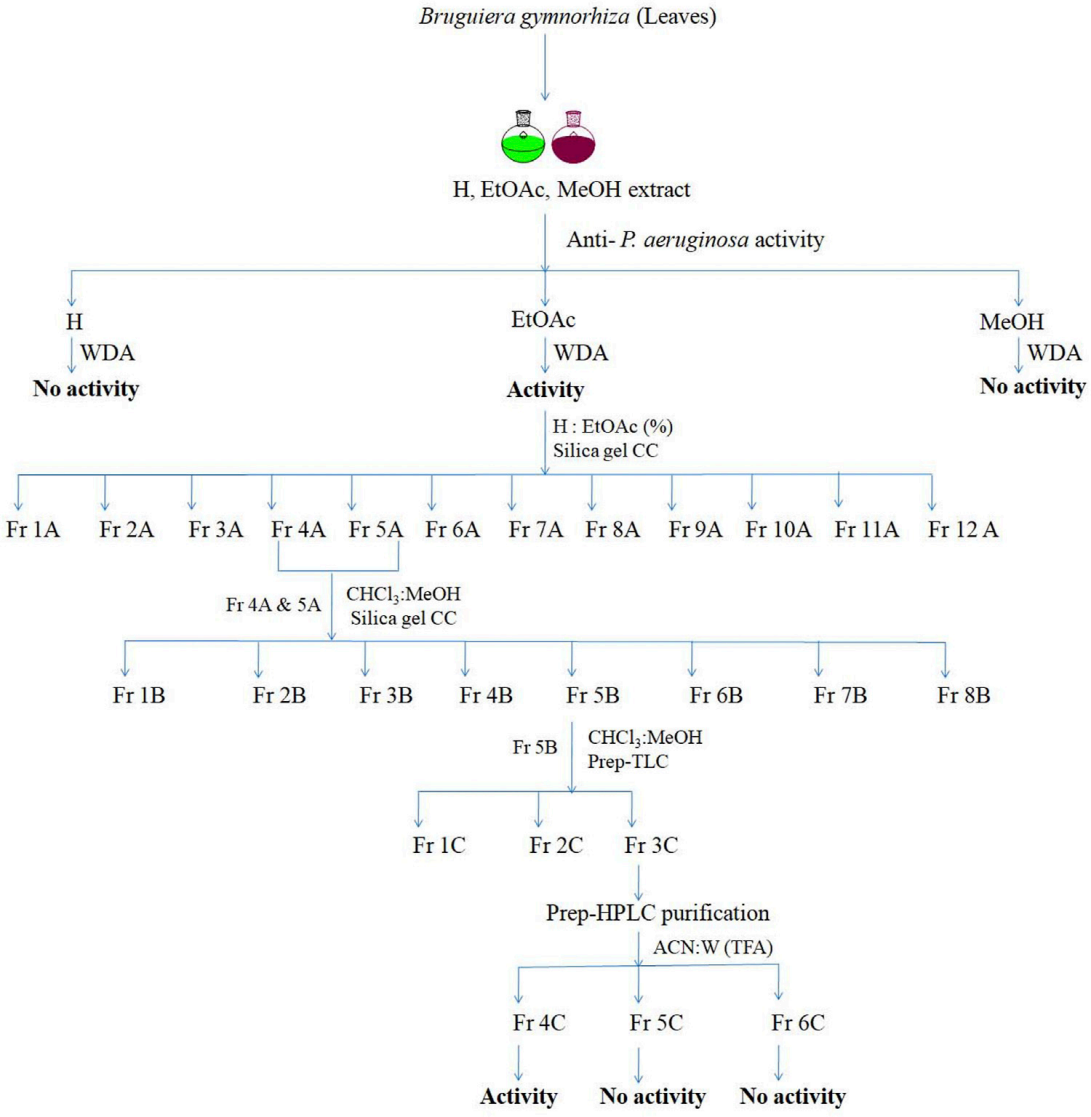
Isolation scheme of active fraction BG138 from leaves of *B. gymnorhiza*. (CC, Column chromatography; H, Hexane; EtOAc, Ethyl acetate; MeOH, Methanol; CHCl3, Chloroform; ACN, Acetonitrile; W, Water; WDA, Well diffusion assay; TFA, Trifluroacetic acid).

The schematic representation of screening of different solvent extracts and the isolation method followed is depicted in a flow diagram (Figure 1). For isolation of active compound, the active crude ethyl acetate extract of leaves (100 gm) was fractionated on a packed silica gel column (800 × 50 mm, 60–120 µm) (10 g extract per 15 g silica) using a mixture of hexane and ethyl acetate (10%, 20%, 30%, 40%, 50%, 60%, 70%, 80%, 90%, and 100% v/v ethyl acetate). A step gradient elution gives 12 total fractions (1A–12A). The antimicrobial active fraction (fraction 4A and 5A, 1 gm) was further mixed and fractionated using a silica gel column (500 × 50 mm, 60–120 µm) by chloroform and methanol mixture (100:0, 99.5:1, 99:1, 98:2, 97:5, 15:1, 10:1, 7:1, 5:1, 3:1, 2:1, and 0:100 v/v), and a total of 225 fractions were collected. Each fraction was analyzed by thin layer chromatography (TLC), and a total of eight pooled fractions (1B–8B) were obtained. Each fraction was tested for activity against *P. aeruginosa*. The active fraction (5B; 225 mg) was further separated using preparative-TLC (20 × 20 cm, Silica gel 60, 254), which yields three sub-fractions (fractions 1C–3C). The fractions were subjected to activity assay using the agar well diffusion method (Balouiri et al., 2016).

The active fraction (3C, 100 mg) was purified through 0.22 nylon filters before separation. The active fraction (3C) was purified on the C18 column (250 mm × 6 mm) using a preparative high-performance liquid chromatography (HPLC) system. A two-component solvent system composed of 0.1% TFA in water (A) and acetonitrile (B) was used as the mobile phase. The chromatographic conditions were 0–5 min, 5% B; 6–10 min, 50% B; 11–20 min, 50% B; 21–25 min, 90% B; and 26–30 min, 5% B, with a flow rate of 1 ml/min. The DAD detector was monitored at 230 nm, which yielded active purified fraction 4C, named “BG138” for further discussion.

#### 2.3.1 Structural Characterization of Bioactive Fraction BG138

The HPLC purified active fraction was analyzed for structural characterization by FTIR (Shimadzu IR Affinity-1S), mass (UHPLC-Q-exactive Orbitrap HRMS system, Thermo Fisher Scientific, Waltham, MA, United States), and NMR spectroscopy (Varian 400 MHz NMR).

### 2.4 Bacterial Strains and Growth Conditions

Bacterial strains used in this study were *Staphylococcus epidermidis* ATCC 12228, *Staphylococcus aureus* MTCC 737, *Streptococcus pyogenes* MTCC 1928, *Acinetobacter baumannii* MTCC 1425, *Klebsiella pneumoniae* MTCC 109, *Escherichia coli* ATCC 25922, *Salmonella enterica* MTCC 1167, *Pseudomonas aeruginosa* MTCC 2582, *P. syringae* DC3000, *P. putida* NCIM 2650, and *Shigella flexneri* MTCC 9543.

*P. aeruginosa* PA2 (MTCC 2582) pure stock cultures were maintained at −80°C in a 25% (v/v) glycerol aqueous solution. 10 μL cultures of *P. aeruginosa* from glycerol stock were plated on the selective Cetrimide agar plate. For all the assays related to *P. aeruginosa*, the working culture was prepared by transferring a single colony to Luria Bertani (LB) broth and incubating at 150 rpm for 18 h at 37°C. In this study, the LB broth was used for most of the assays performed whereas the use of medium other than LB is specified in the respective section.

#### 2.4.1 Growth Curve Analysis of *P. aeruginosa* at Sub-MIC Concentration of BG138

For planktonic cell growth measurement, the assay was performed with some modifications (Huang et al., 2009). For growth measurement assay, overnight-grown culture (OD_620_ nm ≈ 0.5) of *P. aeruginosa* were transferred to 100 ml of LB broth and then supplemented with different concentrations of BG138 (8, 12, and 16 μg/ml). The water (untreated) and DMSO were used as a negative controls. For every 2 h, 100 μL of culture was used to check the turbidity at OD_620_ nm for 18 h using a 96-well microplate spectrophotometer (Thermo Scientific, Waltham, MA, United States). Additionally, at 6, 12, and 18 h of incubation, 100 μL of *P. aeruginosa* suspension culture was serially diluted, and the number of colony forming units (CFUs) per mL of the culture was calculated by plating 10 μL from the resulting dilution on LB agar and incubating at 37°C. The growth of *P. aeruginosa* was expressed in terms of Log CFU/mL.

### 2.5 Determination of Minimum Inhibitory Concentration and Antibacterial Assay

The minimum inhibitory concentration of active fraction BG138 was established using the broth micro-dilution method (CLSI 2015; Wiegand et al., 2008). The inoculum size of the culture used was adjusted to 1 × 10^5^ CFU/ml. The stock solution of BG138 (15 mg/ml) was prepared fresh in dimethyl sulfoxide (DMSO). For the assay, a two-fold dilution of BG138 (4–256 μg/ml) was prepared in recommended Mueller Hinton broth and incubated at 37°C for 18 h. The assay was carried out in triplicates. After incubation, the MIC of BG138 was determined as the lowest concentration which resulted in the inhibition of the visible growth of *P. aeruginosa*. Salicylic acid (SA) was used as a positive control and also evaluated for its minimum inhibitory concentration as mentioned above and used at its sub-MIC concentrations for assays.

In addition to the determination of the MIC against the *P. aeruginosa*, the antibacterial effect of BG138 was also checked against other pathogens (mentioned in Section 2.4) by disc diffusion assay using Mueller Hinton agar as reported earlier (Balouiri et al., 2016). The bacterial cultures were grown freshly by inoculating 10 μL from glycerol stock (stored at −80°C) to 5 ml of Mueller Hinton broth. For the antibacterial assay, the working bacterial inoculums of 1 × 10^8^ CFU/ml were prepared in sterile phosphate buffer saline (pH 7.2). Bacterial suspensions were spread evenly on agar plates, and the sterile disc impregnated with BG138 was placed. Then culture plates were incubated for 24 h at 37°C. The antibiotics (vancomycin, ampicillin, kanamycin, and ciprofloxacin) and DMSO were used as a positive and negative control, respectively. The antimicrobial activities were recorded by measuring the zone of inhibition (Himedia, India).

### 2.6 UPLC-ESI-MS/MS–Based Analysis of the *P*. *aeruginosa* AHLs

The anti-QS capacity of purified fraction BG138 was assessed for quorum sensing inhibitory effect by quantifying C4-HSL and 3-oxo-C12-HSL AHL molecules secreted by *P. aeruginosa* strain (Yang et al., 2018). For estimation of the AHL level of *P. aeruginosa*, 100 μL of culture was added in 50 ml of LB having 12 and 16 μg/ml BG138 and incubated at 37°C for 18 h. After incubation, cultures were centrifuged at 4°C for 10 min, and supernatants were extracted three times using acidified (0.1% acetic acid) ethyl acetate (1:1, v/v). The upper layer was pooled separately and concentrated using a rotary evaporator (30°C). The concentrate was stored in methanol at −20°C. The DMSO was used as the untreated control.

A UPLC system with a 1290 flexible pump equipped with a Triple Quad mass spectrometer (Agilent Technologies, Santa Clara, CA, United States) was employed for analysis. The chromatographic separation was achieved using a Zorbax Eclipse plus C18 column (2.1 × 150 mm, 1.8 μ, Agilent Technology, Santa Clara, CA, United States). For separations, a two-component mobile phase solvent system having aqueous 0.1% formic acid (A) and methanol (B) was used. The injection volume used was 10 μL with a flow rate of 300 μL with a total run time of 22 min. The gradient was set as follows: 1–11 min, 5% B, increase to 50% B in 1 min, and then to 100% B at 18 min. At 19 min, B was reduced to 50% and again set to 5% B till 22 min for re-equilibration. The column and autosampler temperature was stabilized at 30°C and 5°C, respectively. A Triple Quad mass analyzer was used with the electrospray ionization source operating at 350°C. The capillary voltage and nebulizer gas flow were set to 4 kV and 15 psi, respectively. Drying gas was at a flow rate of 10 L/min. The full scan spectrum was recorded from *m/z* 100 to *m/z* 1000. Peaks corresponding to C4-HSL and 3-oxo-C12-HSL analytical standards were detected in positive mode, and their fragmentation behavior and retention time were recorded with the help of published data (Kusar et al., 2016; Yang et al., 2018). Blank injections were used in between sample injections to check and avoid the sample carryover. Product ion *m/z* 102 due to its specificity and signal-to-noise ratio was selected for quantification. The extracted ion chromatogram (EIC) was used for the calculation of peak area, and data were normalized to the level of negative control DMSO to discuss experimental results. Data acquisition and processing were accomplished using Mass Hunter software, Version B.08 (Agilent Technologies, Santa Clara, MA, United States).

### 2.7 Biofilm Inhibition Assay

The effect of BG138 on *P. aeruginosa* biofilm formation was quantified based on a reported method with some modifications using a 96-well microtiter culture plate (Chen et al., 2018). For the assay, 196 μL LB broth and 4 μL of the overnight-grown culture of *P. aeruginosa* were added to each well and supplemented with the different concentrations of BG138 purified fraction to be tested (8, 12, 16, and 20 μg/ml) in the respective wells of the 96-well microtiter culture plate and kept at 37°C for 24 h, without agitation. After incubation, the plates were washed carefully with phosphate buffer saline (PBS, pH 7.0) and dried for 30 min. Then a biofilm was stained with 0.05% (w/v) aqueous crystal violet stain for 15 min. After staining, plates were washed to remove an excess stain with the PBS, and dried and bound crystal violet was solubilized in 0.1 ml of 95% acetic acid. The absorbance was recorded at OD_570_ nm using a microplate reader (Thermo Scientific, Waltham, MA, United States). The biofilm content was normalized to the level of water and DMSO negative controls to estimate the relative level. SA was used as a positive control.

### 2.8 Scanning Electron Microscopic Analysis

Biofilm inhibition assay was carried out as described by Gao et al. (2015), with some modifications. For assay, LB broth in six-well chambered plates with coverslips (diameter 10 mm) and overnight culture of *P. aeruginosa* treated with purified fraction BG138 of different concentrations (8, 12, 16, and 20 μg/ml) were incubated without agitation for 24 h at 37°C. DMSO and SA (10 mM) were used as the negative and positive controls. Coverslips were washed with PBS and dried. For scanning electron microscopy (SEM), coverslips coated with biofilm were fixed using glutaraldehyde (2.5% in PBS, v/v). The attached cells were fixed by osmium tetroxide and dehydrated with graded ethanol (10%, 20%, 30%, 50%, 70%, 90%, and 100%) for 5 min each. The coverslips were freeze-dried, gold-coated, and analyzed by SEM (Quanta 250 FEG).

### 2.9 Flow Cytometry Analysis

To check the effect of BG138 on membrane disruption, flow cytometry analysis was performed with some modifications (Paderog et al., 2020). *P. aeruginosa* was grown for 12 h to get the exponential phase culture. Then the cells were separated by centrifugation (5,000 rpm, 6 min), dissolved in PBS (pH 7.2), and adjusted to a bacterial density of OD_620 nm_ ≈ 0.1. The suspensions were transferred to separate sterile tubes and treated with DMSO, ethanol, and BG138 (32 and 64 μg/ml) and incubated for 4 h at 37°C. DMSO and 70% ethanol-treated cells were used as the negative and positive controls. After incubation, the cells were centrifuged and dissolved in 1 ml of PBS, and 5.9 μL of propidium iodide (PI, Stock:10 mM) was added and kept at 37°C for 15 min (Paderog et al., 2020). After incubation, the cells were centrifuged, suspended in 1 ml PBS, and added to fluorescence-activated cell sorting (FACS) tubes for analysis. The cells were subjected to analysis using the BD FACS Melody cell sorter flow cytometry system (BD, New Jersey, United States). Two thousand cell events were acquired, and PI fluorescence was recorded (642–745 nm). The percentage of PI-stained cells was determined using BD FACS Chorus^TM^ application software. PI fluorescing cells (P8) were gated as dead or membrane-damaged cells, while untreated cells (P7) were gated as live cells (Yasir et al., 2019; Jeyanthi et al., 2021).

Moreover, to evaluate the possible mode of action and cellular effect against *P. aeruginosa*, a morphological analysis was performed following treatment with an active fraction (Goldbeck et al., 2014). The inoculum (2 ml) was adjusted to 1 × 10^5^ CFU/ml and supplemented with BG138 at MIC concentration (32 μg/ml) and further incubated at 37°C for 4 h. A sample for SEM observation was prepared as discussed above (Section 2.8).

### 2.10 Determination of Virulence Factors

The effect of BG138 on virulence factor production was studied at three different concentration levels (8, 12, and 16 μg/ml). The natural QS inhibitor SA (SA, 6 mM) (Ahmed et al., 2019) and DMSO were used as the positive and negative controls, respectively. *P. aeruginosa* was cultured in LB broth and incubated at 150 rpm for 18 h at 37°C. LB broth was used thoroughly for all the virulence factor assays with the exception of pyocyanin and pyoverdine. All the virulence factor levels were normalized and expressed relative to the DMSO control.

The protease activity was determined as reported earlier (Hazen et al., 1965). Sterile supernatant of 150 μL from culture grown over 18 h with different concentrations of BG138 (8, 12, and 16 μg/ml) was mixed with 250 μL of 0.3% azocasein prepared in Tris-HCl (50 mM) and incubated for 4 h at 37°C. Then 1.2 ml of 10% TCA (trichloroacetic acid) was used for precipitation of the substrate which was undigested and centrifuged for 12 min. Afterward, the supernatant was mixed with the same volume of sodium hydroxide (1 M), and the activity was measured at OD_440_ nm.

Pyocyanin and pyoverdine content was estimated using the method (Adonizio et al., 2008; O’Loughlin et al., 2013). For estimation of pyocyanin and pyoverdine, the *P. aeruginosa* was grown separately in 5 ml of broth containing peptone (2%), MgCl_2_ (0.14%), K_2_SO_4_ (1%), and glycerol (1%) and incubated at 37°C for 18 h under shaking. After incubation, the culture was centrifuged (12,000 rpm) for 12 min at 4°C and filtered using a 0.22 μ nylon syringe filter. The cell-free supernatant was used to measure pyocyanin content. Pyocyanin content was measured by absorption at OD_695_ nm. Moreover, for the determination of pyoverdine, filtered supernatant was diluted 10 times in Tris-HCl buffer (pH 7.4) and 100-μL aliquots were transferred to 96-well plates and maintained at low temperature until further analysis. The production of pyoverdine was recorded at OD_405_ nm.

The level of alginate generation was estimated by the addition of 600 μL of boric acid- H_2_SO_4_ (4:1, v/v) to 70 μL of treated and untreated filtered supernatant, and 20 μL of carbazole (0.2%) was added to it. The resulting mixture was kept for 30 min at 55°C, and OD was measured at 530 nm (Zhou et al., 2018).

The orcinol method was employed for the examination of rhamnolipids (Kim and Park, 2013; Rasamiravaka et al., 2015; García-Reyes et al., 2020). The culture was grown for 18 and 24 h in the presence of BG138 (8, 12, and 16 μg/ml). The supernatant (0.3 ml) was extracted with and without overnight precipitation at 4°C using diethyl ether (0.6 ml) and recovered under reduced pressure by evaporating the diethyl ether layer. Subsequently, the residue was stored in 0.1 ml of sterile deionized water. Then, 0.1 ml of each sample was mixed with 0.9 ml of 0.19% orcinol (0.19% orcinol in 53% H_2_SO_4_, v/v) and kept for 30 min at 80°C, cooled, and absorbance was measured at OD_421_ nm.

### 2.11 Motility Inhibition Assays

The effect of BG138 on the swarming and swimming motilities of *P. aeruginosa* was evaluated as reported earlier (Sheng et al., 2015). Briefly, a 3 μL culture of *P. aeruginosa* (OD_620_-0.5) was transferred on a swimming agar plate consisting of 1% tryptone, 0.5% NaCl, and 0.3% agar (pH 7.2) in the absence or presence of BG138 (8, 12, and 16 μg/ml). Similarly, 5 μL of culture was inoculated on the swarming agar plate containing 1% tryptone, 0.5% NaCl, 0.5% glucose, and 0.5% agar (pH 7.2). SA and DMSO served as the positive and negative controls, respectively. Plates were incubated for 24 h at 37°C, and observations were recorded.

### 2.12 Quantitative Real-Time PCR (qRT-PCR)

The effect of BG138 on the expression of QS-related genes was checked using qRT-PCR analysis. Initially, *P. aeruginosa* was grown in LB broth in the presence and absence of BG138 (12 μg/ml) at 37°C for 18 h under continuous shaking conditions. Later, cells were harvested, washed with PBS (pH 7.2), and centrifuged at 4°C. Total RNA was extracted as per the manufacturer’s instructions (VWR Life Science). Complementary DNA (cDNA) was synthesized using the GeneSure first strand cDNA synthesis kit (Genetix Biotech, India). The primers used are listed in Table 1. The qRT-PCR was performed using an SYBR Green qPCR Master Mix (2X Brilliant III SYBR Green qPCR, Agilent Technologies). The *rpsL* served as endogenous control, and the 2^−ΔΔCt^ method was used to determine relative expression changes of the target genes as described previously (Livak and Schmittgen, 2001; Saddhe et al., 2017). Three biological replicates with two technical replicates of each sample were used for analysis*.*

**TABLE 1:** List of primers used in qRT-PCR analysis.

Gene	Primer	Sequence (5'-3')	Number of bases	Amplicon size (bp)
*lasI*	Forward	GGC​TGG​GAC​GTT​AGT​GTC​AT	20	104
	Reverse	AAA​ACC​TGG​GCT​TCA​GGA​GT	20	104
*lasR*	Forward	ACG​CTC​AAG​TGG​AAA​ATT​GG	20	111
	Reverse	TCG​TAG​TCC​TGG​CTG​TCC​TT	20	111
*rhlI*	Forward	AAG​GAC​GTC​TTC​GCC​TAC​CT	20	130
	Reverse	GCA​GGC​TGG​ACC​AGA​ATA​TC	20	130
*rhlR*	Forward	CAT​CCG​ATG​CTG​ATG​TCC​AAC​C	22	101
	Reverse	ATG​ATG​GCG​ATT​TCC​CCG​GAA​C	22	101
*mvfR*	Forward	AAC​CTG​GAA​ATC​GAC​CTG​TG	20	238
	Reverse	TGA​AAT​CGT​CGA​GCA​GTA​CG	20	238
*rpsL*	Forward	GCA​ACT​ATC​AAC​CAG​CTG​GTG	21	231
	Reverse	GCT​GTG​CTC​TTG​CAG​GTT​GTG	21	231

### 2.13 Statistical Analysis

The experimental outcomes are represented as the mean ± standard error. All experiments were performed at least in triplicates. Graphs were generated using OriginPro 9.0 (OriginLab, United States). Statistical difference was determined by one-way analysis of variance (ANOVA), followed by the Tukey-Kramer test, which was used to determine the comparisons between groups. SPSS 15 software (SPSS, Chicago, IL, United States) was used for the statistical analysis. *p* <0.01 is considered as highly significant and labeled as ***, and *p* <0.05 is considered significant and labeled as *.

## 3 Results

### 3.1 Anti–*P*. *aeruginosa* Assay and Determination of Minimum Inhibitory Concentration

Different organic solvents (hexane, ethyl acetate, and methanol) were used to obtain an extract from *B. gymnorhiza* leaves. Among three solvent extracts, only ethyl acetate extract showed potential activity against *P. aeruginosa* PA2. The crude extract was examined for the preliminary determination of the anti–*P. aeruginosa* activity at different concentrations (10, 20, and 30 mg/ml), and the maximum zone of inhibition (26 mm) was recorded at the concentration of 10 mg/ml (Supplementary Information 1, Supplementary Figure S1A). Moreover, the chromatographic fractions were analyzed for anti–*P. aeruginosa* activity by well diffusion and disc diffusion assay, and representative results are depicted in Supplementary Information 1, Supplementary Figures S1B–D. Thereafter, the MIC of purified fraction BG138 was investigated using broth micro-dilution assay in a range of concentrations (8–256 μg/ml). The MIC of BG138 was found to be 32 μg/ml.

**FIGURE 2:**
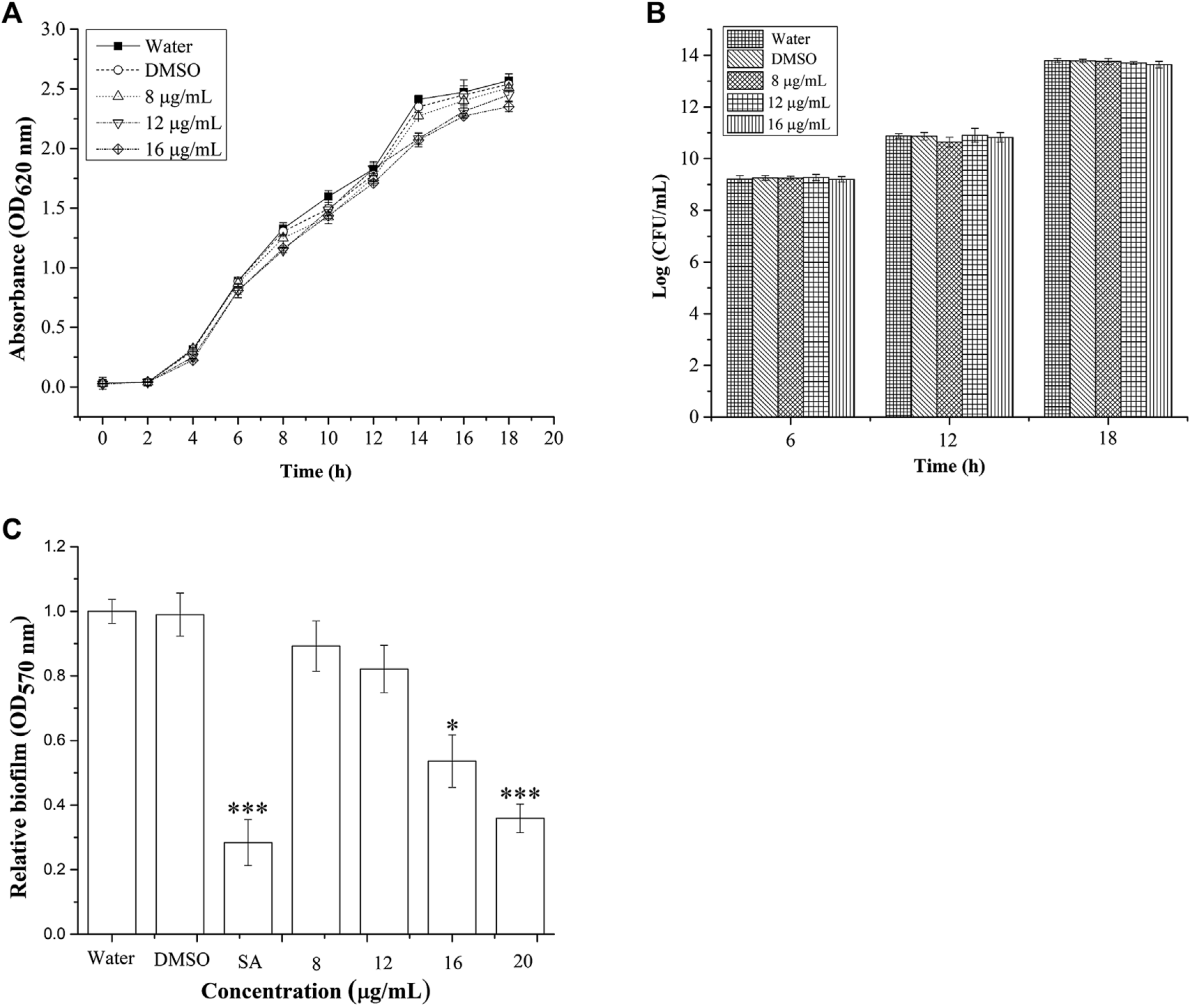
Effect of BG138 on *P. aeruginosa* growth and biofilm. **(A)** Growth was determined for 18 h at different concentrations of BG138 (8, 12, and 16 μg/ml). Water and DMSO served as the negative control. Error bars represent the standard error of three measurements, **(B)** surviving planktonic cells expressed as Log CFU/mL in the culture untreated (water) and treated with DMSO, BG138 (8, 12, and 16 μg/ml), and **(C)** effect of different concentrations of BG138 (8, 12, 16, and 20 μg/ml) on biofilm formation. SA was used as the positive control. Statistical differences were determined by ANOVA followed by Tukey test. *p* < 0.05 (*) *p* < 0.01 (***) versus DMSO control.

The growth of *P. aeruginosa* was monitored for 18 h, at different sub-MIC concentrations (Figure 2). Treatment with concentrations of BG138 varying between 8, 12, and 16 μg/ml showed no drastic inhibitory effect on *P. aeruginosa* cell growth compared to water and DMSO as controls during the stationary growth phase. DMSO was used as a negative control because it enabled easy solubilization of BG138. The DMSO-treated sample was used to find out its effect on untreated cells, and results show that DMSO does not cause reduction in planktonic cells compared to the water control. The growth of *P. aeruginosa* at a concentration of 8 μg/ml BG138 compared to the water and DMSO controls shows no significant difference (*p* <0.05) in comparison to untreated culture (water control). The bacterial growth was also estimated in Log (CFU/ml) at 6, 12, and 18 h incubation time points which suggest BG138 had no significant inhibitory effect on the growth of *P. aeruginosa* over selected concentrations (Figure 2B).

The antimicrobial effect of purified fraction BG138 was evaluated against the other bacterial species such as *Staphylococcus epidermidis* ATCC 12228, *Staphylococcus aureus* MTCC 737, *Streptococcus pyogenes* MTCC 1928, *Acinetobacter baumannii* MTCC 1425, Kle*bsiella pneumoniae* MTCC 109, *Escherichia coli* ATCC 25922, *Salmonella enterica* MTCC 1167, *P. syringae* DC3000, *P. putida* NCIM 2650, and *Shigella flexneri* MTCC 9543, but effective activity in terms of a clear zone of inhibition was not observed (Supplementary Information 1, Supplementary Figure S2). Interestingly, it has been observed that BG138 is selectively active against *P. aeruginosa* with a higher inhibition rate. Therefore, we continued with *P. aeruginosa* for further studies on anti-virulence, anti-biofilm, and cell-damaging action.

### 3.2 Identification of Brugierol/Isobrugierol as Bioactive Compound

The BG138 was analyzed by HPLC, which exhibited the main peak with >96% purity and a few minor contaminants. The molecular mass was estimated by high-resolution mass spectrometry (HRMS) (Supplementary Information 1, Supplementary Figure S3A). HRESIMS *m/z* 138.9870 [M+ H]^+^ revealed the exact mass of 137.9870. FTIR analysis IR (film): *v*_max_ = 3353.7, 2927.8, 1043.2, 1200.2, and 460.3 cm^−1^, and the structures of brugierol and isobrugierol are shown in Supplementary Information 1 (Supplementary Figures S3, S4). The structure was assigned after examining 1D NMR (^1^H, ^13^C, and DEPT experiments) and 2D-NMR (COSY, TOCSY, HSQC, and HMBC experiments) by utilizing a Varian spectrometer with a ^1^H frequency of 400 MHz and a ^13^C frequency of 100 MHz and CDCl_3_ used as a solvent. Homo-nuclear COSY and TOCSY experiments as well as hetero-nuclear HMBC and HSQC experiments revealed the presence of two ring systems. ^1^H chemical shift values at δ (ppm) 5.39 (m, 1H), 3.89 (dd, J = 4.5, 11.4 Hz, 1H), 3.64 (m, 1H), 3.48 (m, 1H), 3.47 (m, 1H), 2.76 (brs, 1H) and ^13^C at δ (ppm) 76.3, 71.1 and 44.0 corresponds to brugierol, while ^1^H at δ (ppm) 5.46 (brs, 1H), 4.55 (m, 1H), 4.11 (m, 2H), 3.62 (m, 1H), 2.93 (dd, J = 3.5, 12.9 Hz, 1H), and ^13^C at δ (ppm) 80.7, 67.3 and 48.5 corresponds to isobrugierol (Supplementary Information 2, Supplementary Figures S1A–F). These chemical shift values are in good accordance with earlier reported values of brugierol and isobrugierol and confirmed the presence of a mixture of brugierol and isobrugierol (Hai-Li et al., 2008; Lopez et al., 2015).

### 3.3 BG138 Shows Reduction in Biofilm Formation

We tested BG138 for concentration-dependent inhibition of biofilm formation of *P. aeruginosa* (Figure 2C). Crystal violet assay was employed to study the effect of BG138 on biofilms in the 96-well microtiter plate. It has been found that there is no significant reduction in inhibition of biofilm formation at a concentration of 8 and 12 μg/ml, whereas with an increase in the concentration of BG138 at 16 μg/ml (*p* < 0.05), the formation of biofilm was gradually reduced by 42%. Additionally, treatment with a concentration of BG138 at 20 μg/ml significantly reduced the biofilm by 61.8% (*p* < 0.01), which is relatively similar to the SA (69.7%). DMSO showed no inhibitory effect toward the biofilm formation compared with the water control.

### 3.4 Effect of BG138 on Quorum Sensing Signaling Molecules (C4‐HSL and C12‐HSL)

**FIGURE 3:**
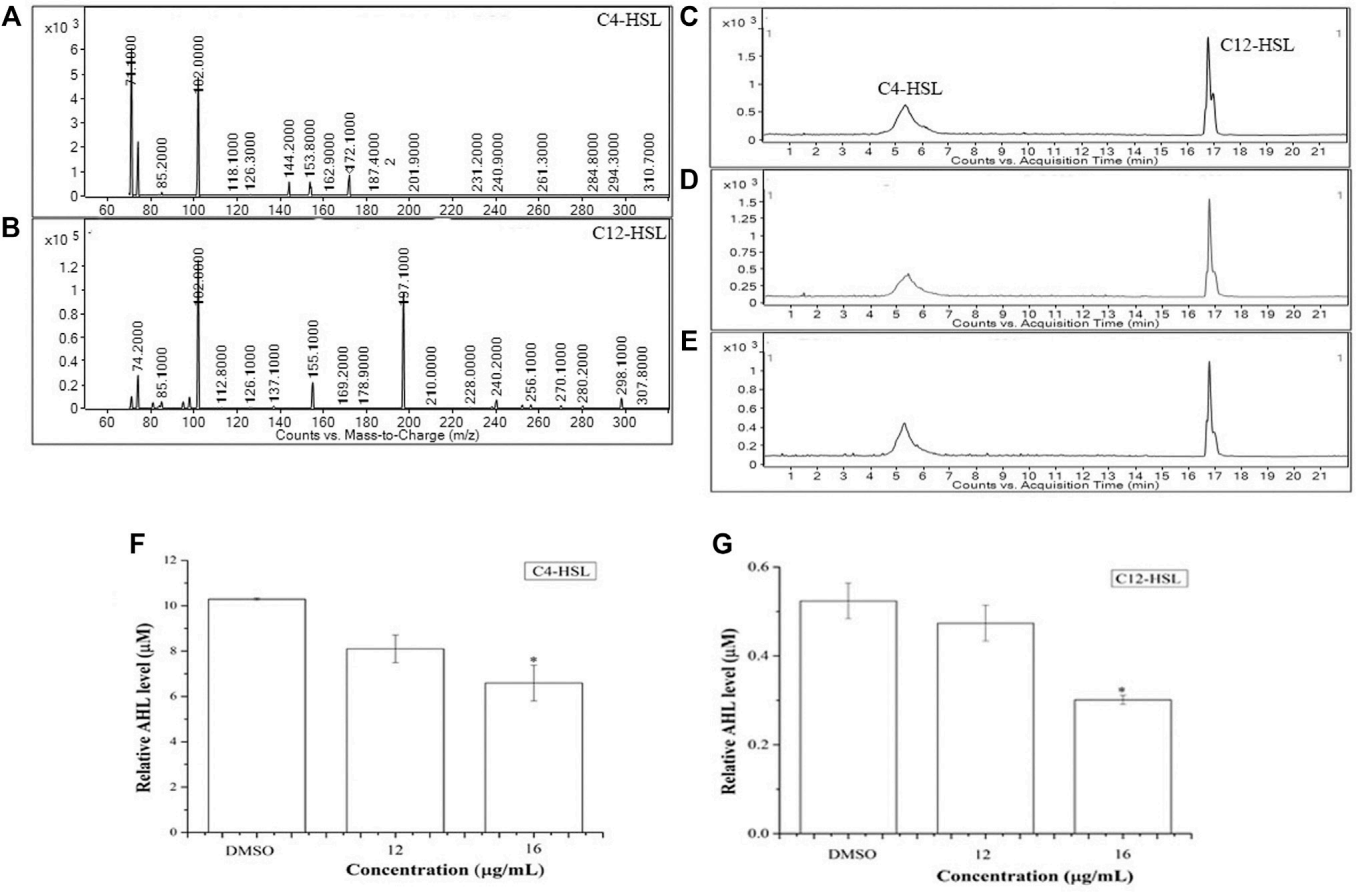
Relative quantification of AHL molecules (C4-HSL and 3-oxo-C12-HSL) in response to BG138, using UPLC-ESI-MS/MS. Product ion spectra of C4-HSL and 3-oxo- C12-HSL **(A,B)**. Chromatograms of C4-HSL and 3-oxo-C12-HSL produced by *P. aeruginosa* treated with DMSO **(C)** and BG138 (12, and 16 μg/ml) **(D,E)**. Quantitative analysis of C4-HSL and 3-oxo-C12-HSL treated with 12 and 16 μg/ml of BG138 **(F,G)**. Error bars are the standard error of three measurements. Statistical differences were determined by ANOVA followed by Tukey test. *p* < 0.05 (*) *p* < 0.01 (***) versus DMSO control.

The possible anti-QS activity of BG138 was checked by examining the change in relative levels of C4-HSL and 3-oxo-C12-HSL from *P. aeruginosa* by UPLC-ESI-MS/MS. The full scan mass spectrum, product ions, and chromatograms are shown in Figure 3. After 18 h of exposure of BG138 at 12 and 16 μg/ml caused a reduction in peak intensity and the peak area of C4-HSL and 3-oxo-C12-HSL (Figures 3C–E). Analysis of relative levels of these AHLs demonstrates that BG138 treatment at 16 μg/ml reduced C4-HSL by 35.9% and 3-oxo-C12HSL by 42.46% (*p* <0.05) in comparison to the control (Figures 3F,G). This demonstrates the anti-QS activity of BG138, which might be due to impeding AHL production.

### 3.5 Scanning Electron Microscopic Analysis

**FIGURE 4:**
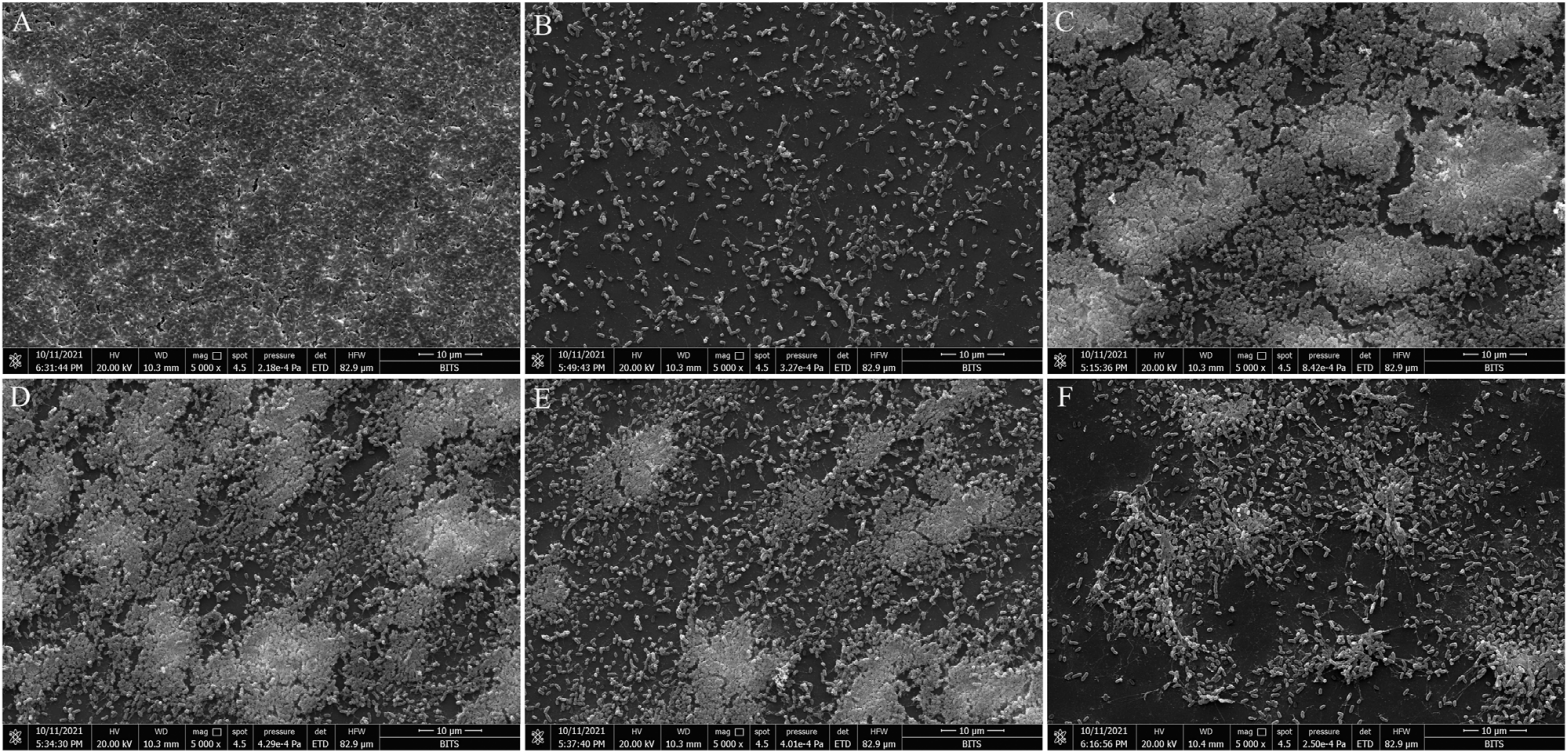
Scanning electron microscopy analysis. SEM images of inhibition of *P. aeruginosa* biofilms treated with DMSO **(A)**, salicylic acid, 10 mM **(B)**, BG138, 8 μg/ml **(C)**, 12 μg/ml **(D)**, 16 μg/ml **(E)**, and 20 μg/ml **(F)**.

Microscopy-based analysis is well known to extract useful information on bacterial morphology and biofilms. Hence, scanning electron microscopy (SEM) was employed to study the effect of BG138 on biofilm. With an exception to other assays, here, 10 mM SA concentration was selected to show its biofilm inhibition effect, as 6 mM concentration was not very effective for consideration as a positive control. In a control experiment, *P. aeruginosa* was treated with DMSO, where a well-developed and compact thick coating of biofilms was observed, whereas 10 mM SA (positive control) showed considerable reduction of biofilm (Figures 4A,B). Treatment with BG138 at 8 μg/ml exhibited no considerable reduction in the biofilm (Figure 4C). As displayed in Figure 4D, with the concentration of BG138 (16 μg/ml), a poorly developed biofilm was observed (Figure 4E). However, BG138 at a concentration of 20 μg/ml showed a notable reduction in the biofilm compared to the control (Figure 4F).

### 3.6 Flow Cytometry Analysis Demonstrates Change in Membrane Permeability of *P*. *aeruginosa*

**FIGURE 5:**
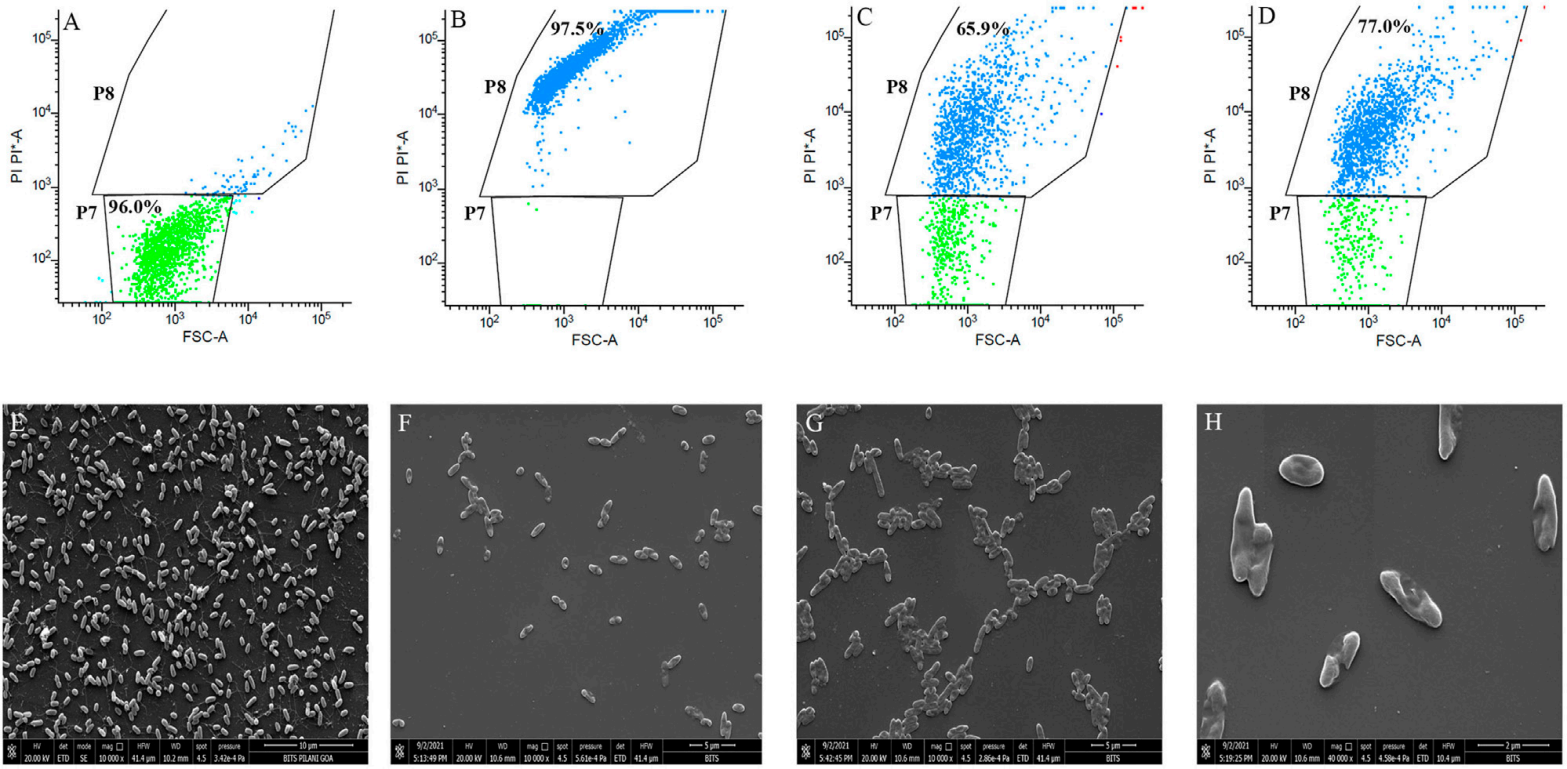
Cell membrane permeability effect of BG138. Data presented as flow cytometer dot plot profile of different treatments: **(A)** DMSO, **(B)** 70% Ethanol, **(C)** 32 μg/ml, and **(D)** 64 μg/ml. SEM images represent the effect of MIC on morphology of *P. aeruginosa,* DMSO **(E)**, MIC **(F–H)**.

To further check the damaging effects of BG138 on *P. aeruginosa* membrane permeability, flow cytometry analysis was performed using propidium iodide (PI), a red-fluorescent dye that can only penetrate damaged or compromised cell membranes (Robertson et al., 2019). The analysis was based on the forward scatter and PI fluorescence signal. Cell population were clustered into two regions, and it is represented on dot plots as P7 and P8 (PI absorption vs. forward scatter). Cells with strong PI fluorescence were regarded as dead or membrane-damaged cells (P8), whereas PI negative cells were considered live cells (P7). Figure 5A shows that untreated cells appeared in the PI negative region (P7) and considered as live cells (98.10%). In contrast, ethanol-treated cells showed the highest cell population (99.6%) with PI fluorescence (Figure 5B), indicating that the cells have damaged cell membranes or are dead cells. After 4 h of exposure, *P. aeruginosa* with a concentration of BG138 of 32 μg/ml (MIC) and 64 μg/ml (2 × MIC) exhibited 65.9% and 77.0% of cells with the damaged cell membrane, respectively (Figures 5C,D). Therefore, the results demonstrate that treatment with BG138 results in increased permeability of the cell membrane.

Additionally, the membrane-damaging activity of BG138 against *P. aeruginosa* was checked by analyzing cell morphology using scanning electron microscopy. Figures 5E–H shows untreated cells with intact cell membrane, and treated cells with changed morphology were observed after exposure to BG138. Results revealed that exposure of *P. aeruginosa* to the BG138 (32 μg/ml) showed destruction of the cells and change in the morphology of the cell membrane, which might be due to the membrane-damaging activity of BG138.

### 3.7 BG138 Shows Reduction in *P. aeruginosa* Virulence Factors

**FIGURE 6:**
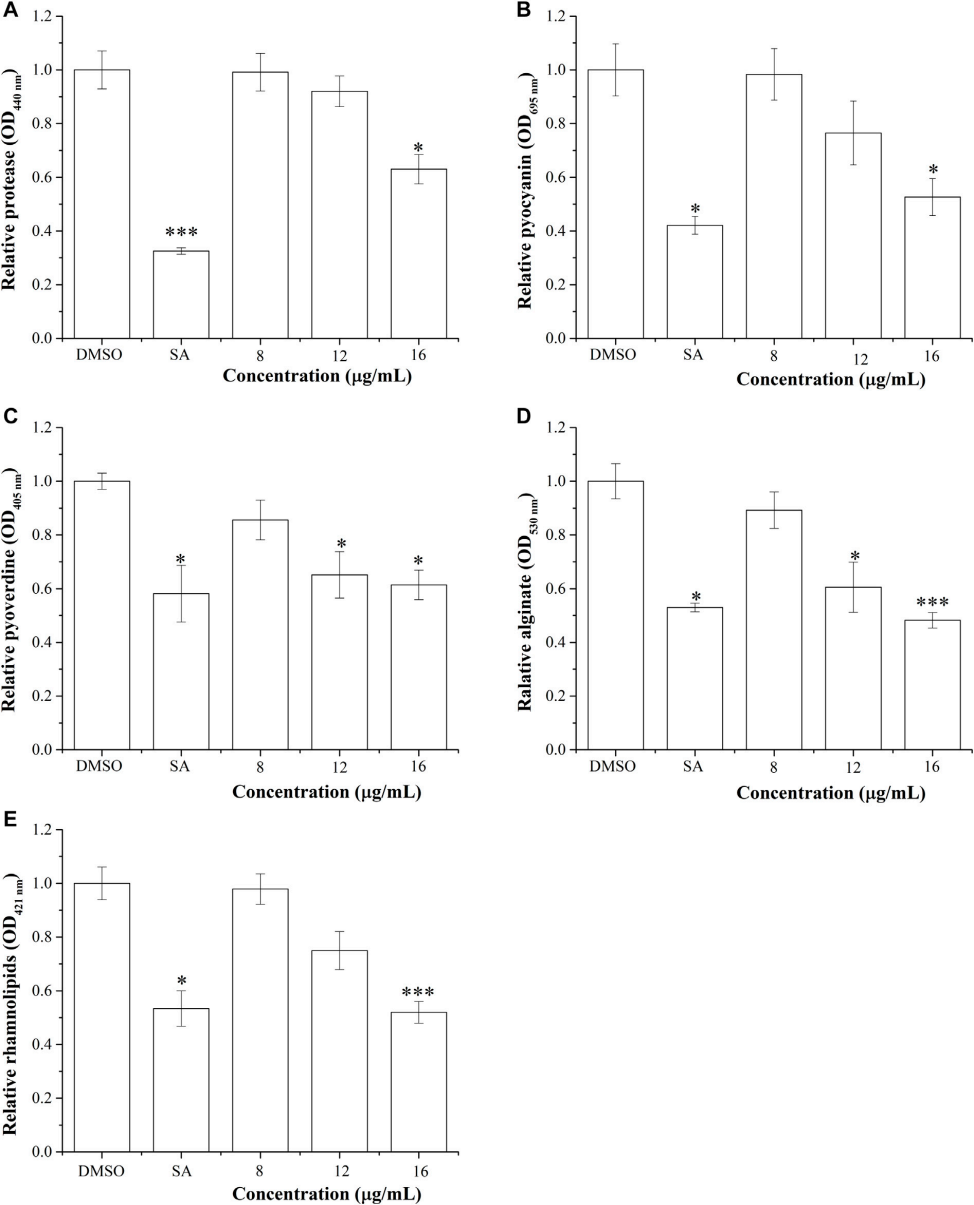
Effect of BG138 on virulence factors secreted by *P. aeruginosa* PA2. Relative levels of virulence factors at different BG138 concentrations (8, 12, and 16 μg/ml) were tested. DMSO and salicylic acid were used as negative and positive controls. **(A)** protease levels, **(B)** pyocyanin levels, **(C)** pyoverdine levels, **(D)** alginate levels, and **(E)** rhamnolipid levels. Statistical differences were determined by ANOVA followed by Tukey test. *p* <0.05 (*) *p* <0.01 (***) versus DMSO control.

The virulence factors are the molecules that help bacteria in colonization and survival. Here, we have studied the effect of BG138 on the virulence factors of *P. aeruginosa*. Results of respective assays are depicted in Figures 6A–E. As indicated in Figure 6A, the relative level of the protease was reduced by treatment of BG138 over selected concentrations (8, 12, and 16 μg/ml). Treatment with 8 and 12 μg/ml of BG138 did not result in significant inhibition. At an increased concentration of 16 μg/ml of BG138, there was a significant inhibition of protease activity by 36% (*p* <0.05), whereas SA showed inhibition by 67% (*p* <0.01).

The depletion in the production of pyocyanin after exposure to active metabolites is also an indicator of its QS-inhibitory nature in *P. aeruginosa.* The production of pyocyanin is regulated by *rhl*-QS. We have tested the effect of different concentrations of BG138 on the production of pyocyanin. Assay results show that the relative level of pyocyanin decreased by 23% and 47% on exposure to BG138 at concentrations of 12 and 16 μg/ml (*p* < 0.05), respectively (Figure 6B). Similarly, different concentrations of BG138 were examined to study its effect on pyoverdine production. As shown in Figure 6C, a notable reduction of pyoverdine level by 34% and 38% was observed at 12 μg/ml (*p* <0.05) and 16 μg/ml (*p* <0.05) of BG138. Furthermore, SA showed nearly 41% inhibition.

The alginate is one of the important components of the extracellular matrix of *P. aeruginosa* (Mahajan et al., 2021). The inhibitory effect of BG138 on alginate production of *P. aeruginosa* was investigated. The result demonstrated a significant reduction in alginate production at the concentrations of 12 μg/ml (*p* <0.05) and 16 μg/ml (*p* <0.05) of BG138 (Figure 6D). An approximately 51% reduction of alginate was observed with exposure of 16 μg/ml BG138 treatment. Moreover, the inhibitory effect of different concentrations of BG138 on the rhamnolipid levels was shown in Figure 6E. The reduction in rhamnolipids was found to be concentration driven, causing a reduction by approximately 21%, 25%, and 48% (*p* <0.01) in the rhamnolipid level at concentrations of 8, 12, and 16 μg/ml of BG138 compared with the DMSO control. SA treatment resulted in the reduction of the rhamnolipid level by 46%. In addition to this, rhamnolipids were extracted and estimated with and without overnight precipitation at 4°C, and the effect of BG138 was also tested for 24 h, where no significant difference in the level of rhamnolipids was observed compared to 18 h. The relative levels of rhamnolipids are shown in Supplementary Information 1, Supplementary Figures S5A,B. Additionally, the effect of BG138 on all the virulence factors was studied for 72 h of incubation time, and the result shows no significant difference between the control and treatments, except rhamnolipids (*p* <0.05) (Supplementary Information 1, Supplementary Figure S6).

### 3.8 BG138 Causes Reduced Swimming and Swarming Motility of *P. aeruginosa*

**FIGURE 7:**
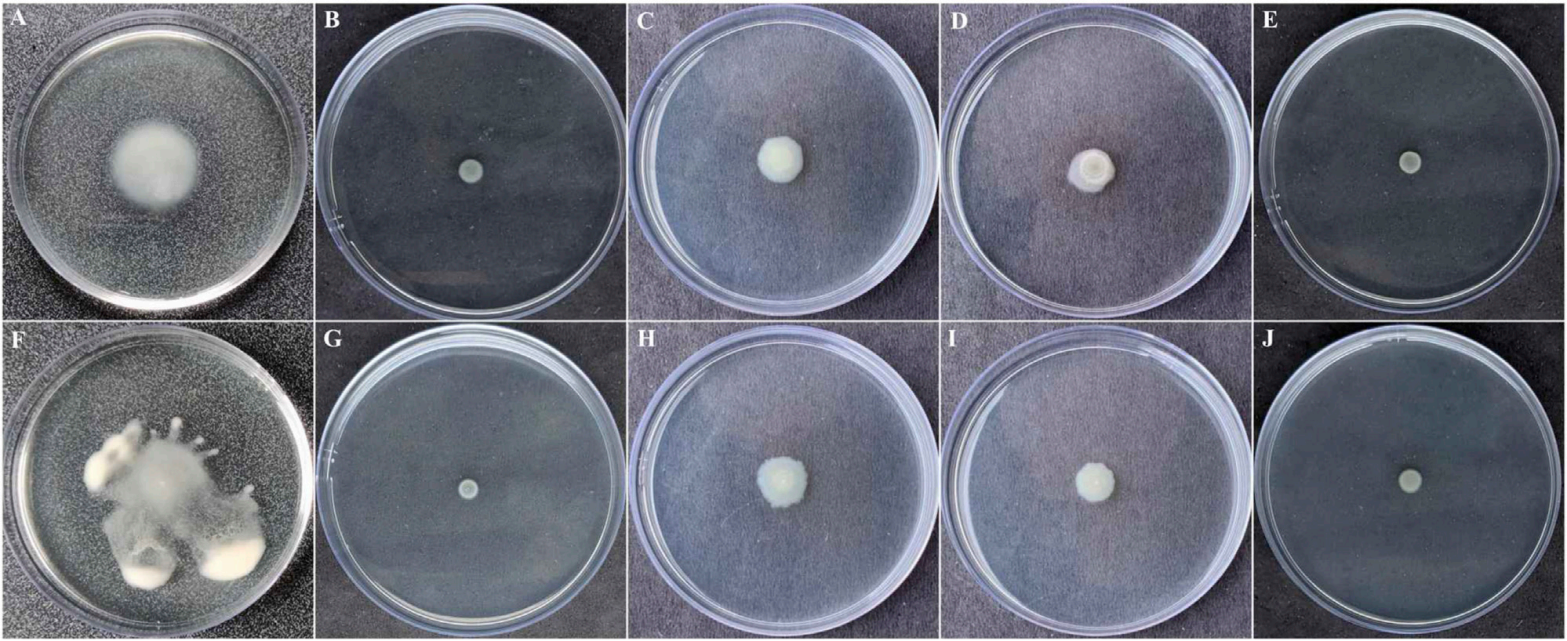
Swimming **(A–E)** and swarming motilities **(F–J)**. Different treatment given DMSO **(A,F)**, and salicylic acid **(B,G)** as positive control and BG138 varying concentrations 8 μg/ml **(C,H)**, 12 μg/ml **(D,I)**, and 16 μg/ml **(E,J)**.

Pathogenesis and QS-directed biofilm formation of *P. aeruginosa* was reported to be dependent on the swarming and swimming motilities (Wadhwa and Berg, 2021)*. P. aeruginosa* were evaluated in the presence of BG138 on swimming and swarming motility studies (Figures 7A–J **)**. For swimming motility studies, treatment with BG138 at varying concentrations (8, 12, and 16 μg/ml) showed a reduction in the colony diameter. Interestingly, treatment with 16 μg/ml of BG138, the colony diameter was reduced from 11 to 7 mm (Figure 7E). Furthermore, in swarming motility assay, treatment with BG138 demonstrated a significant reduction in colony size and tendril formation and a notable reduction in the colony diameter in response to 16 μg/ml of BG138 in comparison with the control (18 mm). Additionally, the effect of SA was also observed. Salicylic acid showed a similar effect to that of BG138 concentration (Figures 7B,G), whereas there was no inhibitory effect found with the DMSO control on motility type (Figures 7A,F).

### 3.9 QS-Related Genes Were Downregulated by Treatment With BG138

**FIGURE 8:**
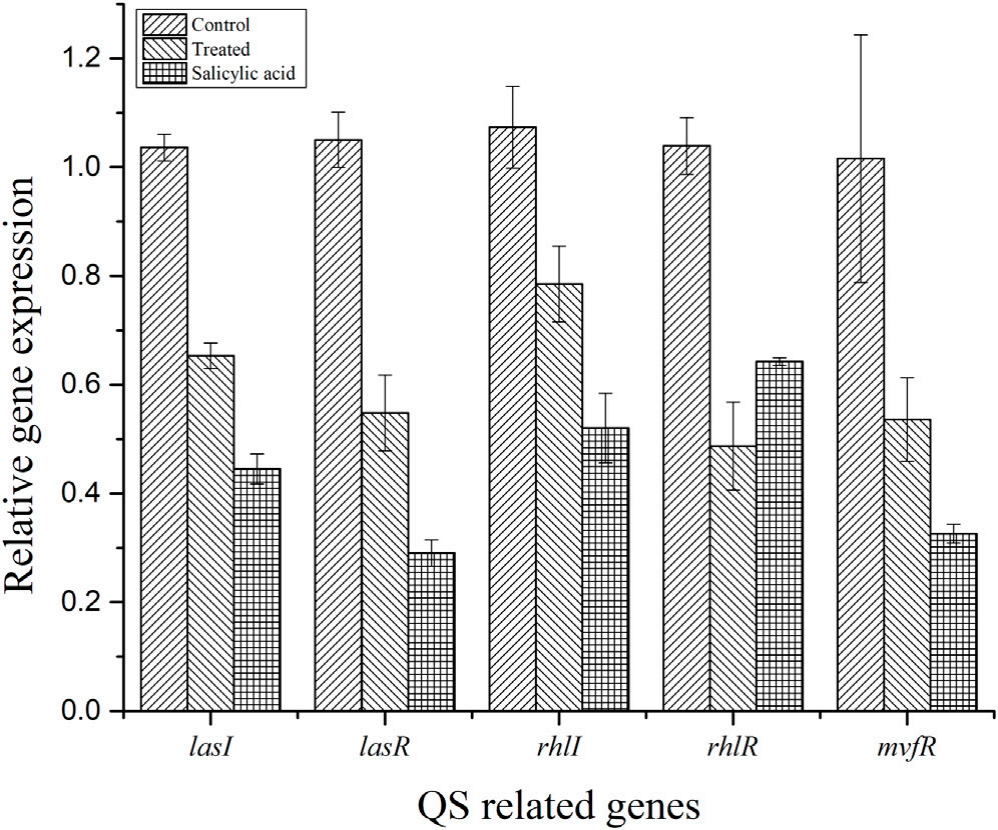
Effect of BG138 (12 μg/ml), on the selected QS-related gene expressions (*lasI*, *lasR*, *rhlI*, *rhlR*, and *mvfR*) of *P. aeruginosa* PA2. DMSO and SA were used as controls.

The efficiency of BG138 on the transcriptional level of five QS-related genes (*lasR*, *lasI*, *rhlR*, *rhlI*, and *mvfR*), encoding biofilm formation and QS factors in *P. aeruginosa* was evaluated by qRT-PCR in 18-h-old culture with BG138 and DMSO as the control (Figure 8). The expression of genes encoding the QS system was decreased significantly when exposed to BG138 at the concentration of 12 μg/ml. The most significant inhibition was found in the case of *rhlR*, followed by *lasR* which was downregulated by approximately 53.10% and 47.82%, respectively, on treatment with BG138. Moreover, the expression of other genes such as *lasI*, *rhlI*, and *mvfR* was also decreased significantly on exposure to BG138*.*

## 4 Discussion

Natural products are widely studied for their bioactive properties. For extraction of metabolites from different parts of plants, different solvents can be used (from non-polar to polar). In this research work, three organic solvents (hexane, ethyl acetate, and methanol) were employed in the initial screening of *B. gymnorhiza* leaves against various human pathogens. Among the selected solvents, ethyl acetate was found to be a suitable solvent for the extraction of anti–*P. aeruginosa* metabolites.

*P. aeruginosa* is a comprehensively studied human pathogen known for causing recurring and chronic infections through a quorum sensing mechanism. Biofilms produced by microbial communities are mainly responsible for the resistance toward existing antibiotics. As a result, it is essential to look for active chemicals in natural sources that can reduce *P. aeruginosa* pathogenicity. The *B. gymnorhiza* and its associates have been reported to possess various biological properties of therapeutic importance such as antimicrobial, antioxidant, neuroprotective, and so on (Wu et al., 2015; Barik et al., 2013). Anti-QS and anti-biofilm compounds such as synthetic AHL analogs (Moore et al., 2015; Shin et al., 2019), emodin, patulin from *Plectosphaerella cucumerina* extract (Zhou et al., 2017), *Berginia ciliata* (Soukarieh et al., 2018), *Clematis viticella* (Alam et al., 2020), *Origanum vulgare* (Camele et al., 2019), and parthenolide (Lu et al., 2019) from natural sources have been reported recently. However, *B. gymnorhiza* has not been evaluated or reported yet as a source of anti-biofilm and anti–quorum sensing activity against *P. aeruginosa.* The structure of the BG138 purified fraction was elucidated by comparing the IR, molecular mass, and 1D and 2D NMR spectroscopic data (Supplementary Information 2, Supplementary Figures S1A–F). The FTIR analysis of a BG138 reveals a peak at 3352.7 cm^−1^ designated O-H group stretching, and the S-S stretch was identified by signal at 447.3 cm^−1^. The stretching vibrations of S-S bonds in FTIR analysis were confirmed from the reported studies (Trofimov et al., 2009). By detailed analysis of 1D and 2D NMR data, the chemical shift values were assigned and then compared with previously published data. These chemical shift values were in good accordance with earlier reported values of brugierol and isobrugierol (Hai-Li et al., 2008; Lopez et al., 2015) and suggested that the bioactive fraction BG138 is the mixture of two already reported cyclic disulfides such brugierol and isobrugierol, which could be responsible for the anti-virulence and anti–quorum sensing activities of *B. gymnorhiza* bioactive fraction. The brugierol/isobrugierol has been previously reported to be found in mangroves, such as *Pelliciera rhizophorae, Bruguiera gymnorhiza*, and *Bruguiera conjugata* (Kato and Numata, 1972; Homhual et al., 2006; Huang et al., 2009; Lopez et al., 2015). In accordance with the earlier report, nearly half of all available drugs are in use as a racemic mixture or in the form of different isomers. Generally, physical or chemical properties of these molecules can differ, and consequently, stereochemistry of the metabolite shows an impact on the difference in the biological action. Hence, metabolites with different isomers can differ in terms of either less or more activity toward pathogens (Hutt and O’Grady, 1996; Tessema et al., 2013; Demir-Ordu et al., 2015). So far, in our study, we have not achieved complete separation of these two diastereomers due to the lack of a sufficient amount of sample, and hence, the entire study was conducted based on a mixture. These diastereomers have not been previously investigated for anti–*P. aeruginosa* bioactivity, aside from anticancer and enzyme inhibitory activity. Moreover, the minimum inhibitory concentration against *P. aeruginosa* reported in our study is lower than those in the earlier reports from natural sources (Latha et al., 2010; Zhou et al., 2017; Zhou et al., 2018). On the basis of our initial antimicrobial assay results, we decided to check BG138 for its effect on QS-related gene expression, QS-controlled virulence, and biofilm formation or associated traits.

Considering the global situation with increasing multi-drug resistance stains, it is desirable to search for different quorum sensing inhibitory metabolites (Hoiby et al., 2010). It is crucial to evaluate the effect of the metabolite to be studied on the growth of *P. aeruginosa* prior to its functional characterization at the level of anti-QS and anti-virulence effect. Various studies have reported the effect of active compounds on *P. aeruginosa* growth at different sub-MIC concentrations (Zhou et al., 2017; Zhou et al., 2018; Rajkumari et al., 2019). Zhou et al. (2017) showed inhibition of *P. aeruginosa* growth at the sub-MIC concentration of 1 mg/ml (MIC, 1.25 mg/ml) of *Plectosphaerella cucumerina* extract. Similarly, in our studies, we found a significant difference in the growth of *P. aeruginosa* after 18 h at treatment 16 μg/ml compared to the DMSO control. It is well known that QS is an intracellular communication system that functions *via* a dedicated secreted signaling molecule for coordinating cell growth and group behaviors in normal and stress conditions (Kostylev et al., 2019; Pena et al., 2019). According to (Diggle et al., 2007; Rutherford and Bassler, 2012), reduction in the level of QS signals (AHL) shows alteration of virulence, gene expressions, and cell population of the organism. Likewise, we also noted the reduction in AHL level by more than 35% at 16 μg/ml of BG138. Therefore, it may be concluded from our study that the difference in cell density at BG138 (16 μg/ml) may be due to the effect of BG138 on the reduction of QS signaling AHL molecules.

As reports suggest, AHL signals of *P. aeruginosa* play a major role in regulation and coordination for the expression of traits responsible for infection and disease, such as virulence factors and biofilm production. Biofilm formation is controlled by QS signaling systems that act as a barrier to resist active metabolites’ passage into cells and aid in its survival under drug exposure (Pena et al., 2019). AHLs of *P. aeruginosa* can interact with the *lasR/rhlR* receptor and trigger QS-related gene expressions responsible for the production of virulence factors and biofilm. The *las* system utilizes 3-oxo-C12-HSL, whereas the *rhl* system uses C4-HSL as a QS signal (Kostylev et al., 2019). Presently, the BG138 treatment shows a remarkable reduction in AHL levels and biofilm inhibition. It shows a significant decrease in AHLs (Figure 3F,G) in *P. aeruginosa* on treatment with BG138 (16 μg/ml), and surprisingly, a characteristic reduction of biofilm at the same concentration of BG138 was also observed (Figure 2C).

In recent years, the flow cytometry technique has become an important tool to test the effect of bioactive molecules on pathogens by fluorescent probes (Duquenoy et al., 2020) and has also been reported as an emerging next-generation tool for antimicrobial susceptibility testing (Van and Dunne, 2013). Girard et al. (2019) and Wu et al. (2016) studied the viability of *E. coli* and *S. aureus* with essential oils and hydroquinic acid. Similarly, the effect of naturally isolated hydroquinone against cell membrane integrity of *P. aeruginosa* and *S. aureus* strains was evaluated by flow cytometry, and more than 46% inhibition of *P. aeruginosa* and *S. aureus* cells after 2 h incubation was reported (Jeyanthi et al., 2021). Likewise, in the current study, the cell viability assay of *P. aeruginosa* was carried out at two concentration levels (1x MIC and 2x MIC), which shows concentration-dependent reduction of the cell population within 4 h. On the other hand, change in morphology and destruction of the cell membrane is demonstrated at the same MIC concentration by SEM analysis which shows irregularly shrunken cells and deformed, damaged membranes after treatment with BG138 for 4 h. Consistent with these results, *Vernonia cinerea* extract against *P. aeruginosa* (Latha et al., 2010) and a semi-synthetic antimicrobial compound such as “3-(p-chlorophenyl)thio citronellal” show morphological changes in *Listeria monocytogenes* and *P. fluorescens* after exposure (Goldbeck et al., 2014). Overall, flow cytometry and SEM analysis confirm enhanced membrane permeability due to deformed cell membranes which may have resulted in increased PI uptake and loss of viability of *P. aeruginosa* when treated with BG138.

Moreover, the suppression of QS-mediated extracellular virulence factor production in *P. aeruginosa* in the presence of BG138 pure fraction shows an indication of its anti-QS property. We investigated the effect of BG138 on the level of protease, pyocyanin, pyoverdine, rhamnolipids, and alginate and found a significant shift in their relative levels when subjected to different concentrations of BG138 (Figures 6A–E). Similarly, studies based on metabolites such as 5-hydroxymethylfurfural (Rajkumari et al., 2019) and endophytic fungus (*Daldinia eschscholtzii*)-derived 2,4-di-tert-butylphenol (Mishra et al., 2020) show a significant reduction in the level of pyocyanin and protease of *P. aeruginosa*. Moreover, a study using plant-derived hordenine molecule has shown concentration-dependent inhibition of production of pyoverdine and other virulence factors (Zhou et al., 2018). As observed in the present study, a significant decrease in protease, pyocyanin, and pyoverdine levels were recorded with 16 μg/mL of the BG138 treatment. Alginate is one of the crucial parts of the bacterial extracellular matrix and is responsible for conserving the biofilm form and blocks entry of active molecules into cells. In addition to the reduced alginate level (Figure 6D), the loosened architecture of biofilms was also observed by SEM on exposure to BG138 (Figures 4E,F). As previously stated, the rhl system controls rhamnolipid synthesis, which is crucial for biofilm development and maintenance in *P. aeruginosa* (Pearson et al., 1997). Furthermore, colonization, biofilm, and virulence are all linked to motility and are thought to have a role in pathogenesis (Badal et al., 2021). We found a significant reduction in rhamnolipids, as well as swimming and swarming motility, in the presence of BG138 (Figures 7E,J).

Furthermore, we utilized qRT-PCR analysis to check the expression of some key QS-related genes like *lasR/lasI, rhlR/rhlI*, and *mvfR* in *P. aeruginosa*. It is well investigated that the *las* and *rhl* system is important for the formation and attachment of biofilm and regulation of biofilm formation. Several virulence factors, including proteases, exotoxin A, and *RhlR* expression, have been found to be regulated by the *las* QS system, whereas the *rhl* QS system has been shown to govern the expression of genes coding for pyocyanin, protease, and rhamnolipids (Glessner et al., 1999). As a result, inhibiting the *las* QS system may certainly affect the *rhl* QS systems. Furthermore, earlier results suggest that *las* deficient *P. aeruginosa* can also probably form biofilms and still present residual virulence (Fong et al., 2019; Soto-Aceves et al., 2019). These results show that the inhibition of lasR or rhlR is not the only way to block *P. aeruginosa* virulence, due to the complexity and regulation of QS at various levels (Ciofu and Tolker-Nielsen, 2019; Fong et al., 2019). In this study, transcriptional levels of QS-related genes (*lasI, lasR, rhlI, rhlR*, and *mvfR*) were reduced by 32–47%, compared with that of the control (Figure 8). This demonstrates that a little reduction in the QS-related gene is sufficient to lower virulence to some extent, as discussed in this study.

There have been few recent studies on the bioactivity potential of brugierol/isobrugierol against microorganisms, and commercial standards for further validation of the study are not currently available. In contrast, we have less opportunity to compare and validate our results with those of other studies using brugierol/isobrugierol as the main metabolite. As a result, we had no choice but to compare it to the anti-QS effects of active metabolites obtained from other plants. In the last decades, garlic oil, aqueous and solvent (methanol and ethanol) extracts, and sulfur-containing components of garlic have been extensively studied for their antibacterial actions, as well as anti-quorum sensing and anti-virulence properties (Lee et al., 2012; Bhatwalkar et al., 2021). The literature shows that sulfur-containing compounds like allicin, ajoene, diallyl trisulfide, and diallyl disulfide form disulfide bonds with free sulfhydryl groups of proteins/enzymes and affect the integrity of the cell membrane or cell wall of bacteria. Besides, sulfur-containing compounds from garlic also show a global effect at the level of DNA replication, translation, and protein synthesis (Ross et al., 2001; Velliyagounder et al., 2012; Bhatwalkar et al., 2021). To date, the relationship between quorum sensing and biofilm formation has not been fully revealed and clarified. As proposed recently (Yasir et al., 2018), biofilm inhibition and disruption by active compounds involve cell death, inhibition of motility, and the enzymes involved in production and secretion of the extracellular matrix, but the precise and complete mechanisms are yet to be elucidated (Srinivasan et al., 2021). Thus, we postulate that BG138 (brugierol/isobrugierol) might be interfering with the synthesis of AHLs and can lead to the inhibition of biofilm and reduction in virulence. Nevertheless, our study shows that the *B. gymnorhiza* may be the reservoir of active antibacterial metabolites for therapeutic use. Therefore, more research is needed to check the bioactivity of brugierol and isobrugierol separately in its purified form. In addition to this, hereafter, it is an ensuing assignment to separate and search the most possible mechanism of action of brugierol and isobrugierol against *P. aeruginosa*.

## 5 Conclusion

In conclusion, this research represents a bioactive fraction BG138 from *B. gymnorhiza* not only significantly attenuated to QS-controlled virulence biofilm formation but also showing changes in cell morphology and membrane damage of *P. aeruginosa*. This is the first report on the anti-QS and anti-biofilm potential of bioactive fraction from *B. gymnorhiza* against *P. aeruginosa*. Furthermore, BG138 shows inhibitory activity toward *P. aeruginosa* and was also found to inhibit the biofilm formation by reducing the motilities and quorum sensing inducers such as C4HSL and C12-HSL. Additionally, it also reduced the level of virulence factors investigated in the current study. The study suggested that BG138 may be interfering with the component of the *P*. *aeruginosa* QS system, which results in a decrease in transcriptional levels of the genes responsible for quorum sensing.

## Data Availability

The original contributions presented in the study are included in the article/Supplementary Materials, further inquiries can be directed to the corresponding author.
