# Supplementary material for: A Cyclic Disulfide Diastereomer From Bioactive Fraction of Bruguiera gymnorhiza Shows Anti–Pseudomonas aeruginosa Activity
Source: Front Pharmacol. 2022 Jun 2;13:890790. doi: 10.3389/fphar.2022.890790 (PMC9201687; doi:10.3389/fphar.2022.890790)

Supplementary information 2

Figure S1: One-dimensional (1D) and two-dimensional (2D) NMR analysis of BG138.
(A) 1H NMR, (B) 13C NMR, (C) COSY, (D) gHSQC, (E) gHMBC, and (F) DEPT_45, 90, 135 spectrum

(A) 1H NMR

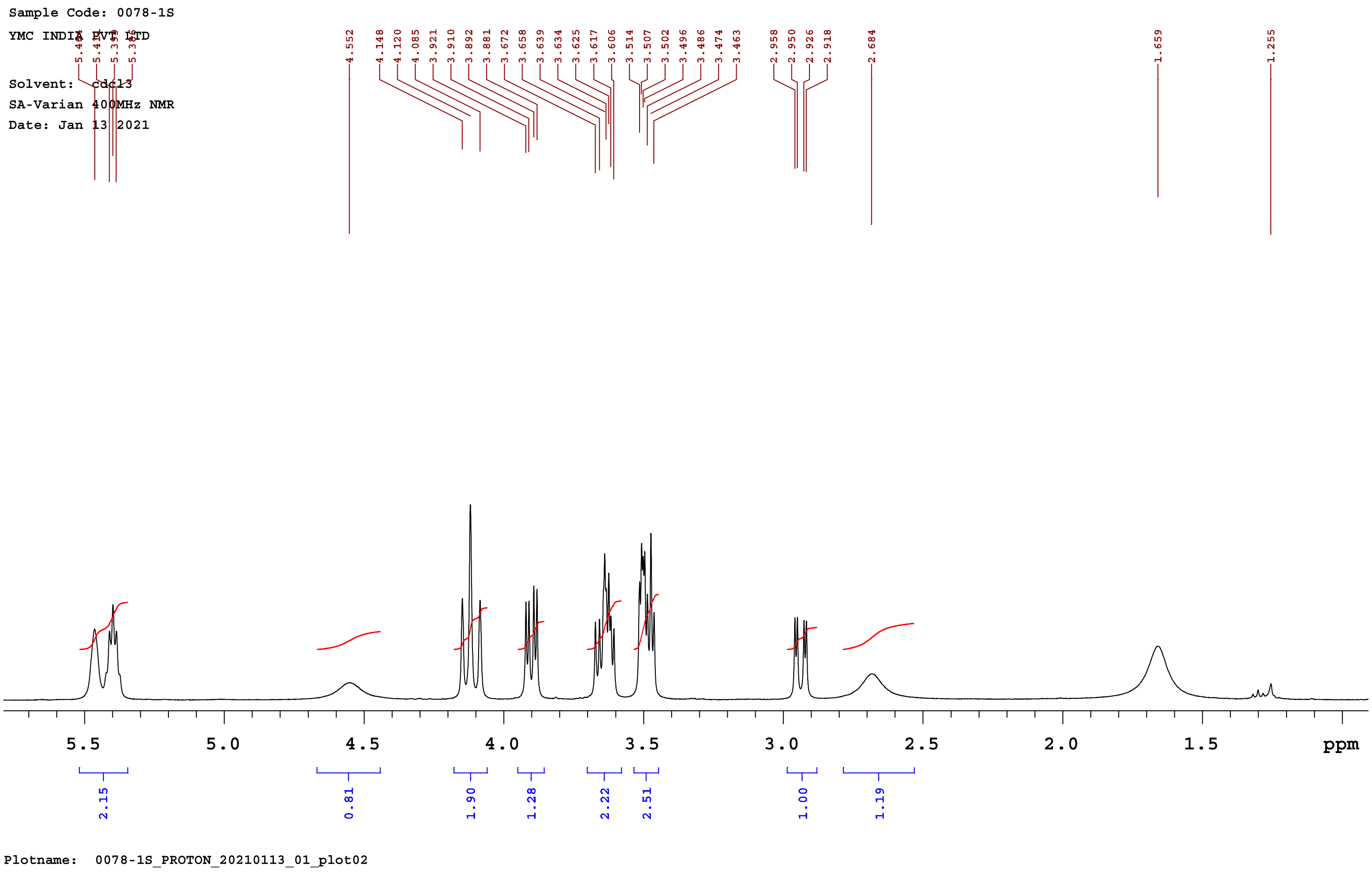

(B) 13C NMR

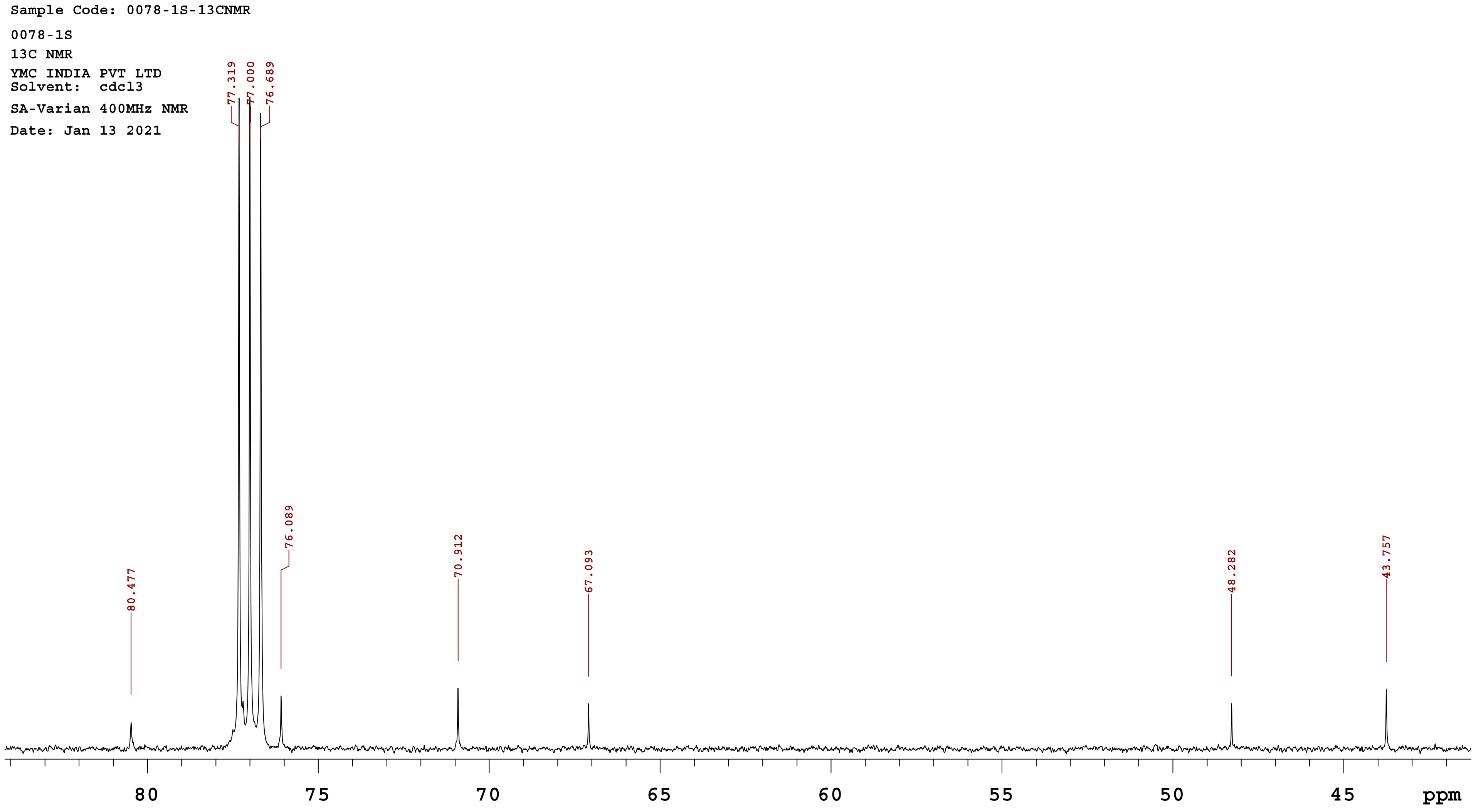

Sample Code: 0078-1S-13CNMR
0078-1S
13C NMR
YMC INDIA PVT LTD
Solvent: cdcl3
SA-Varian 400MHz NMR
Date: Jan 13 2021
77.319
77.000
76.689
76.089
80.477
70.912
67.093
48.282
43.757
80
75
70
65
60
55
50
45
ppm

(C) COSY

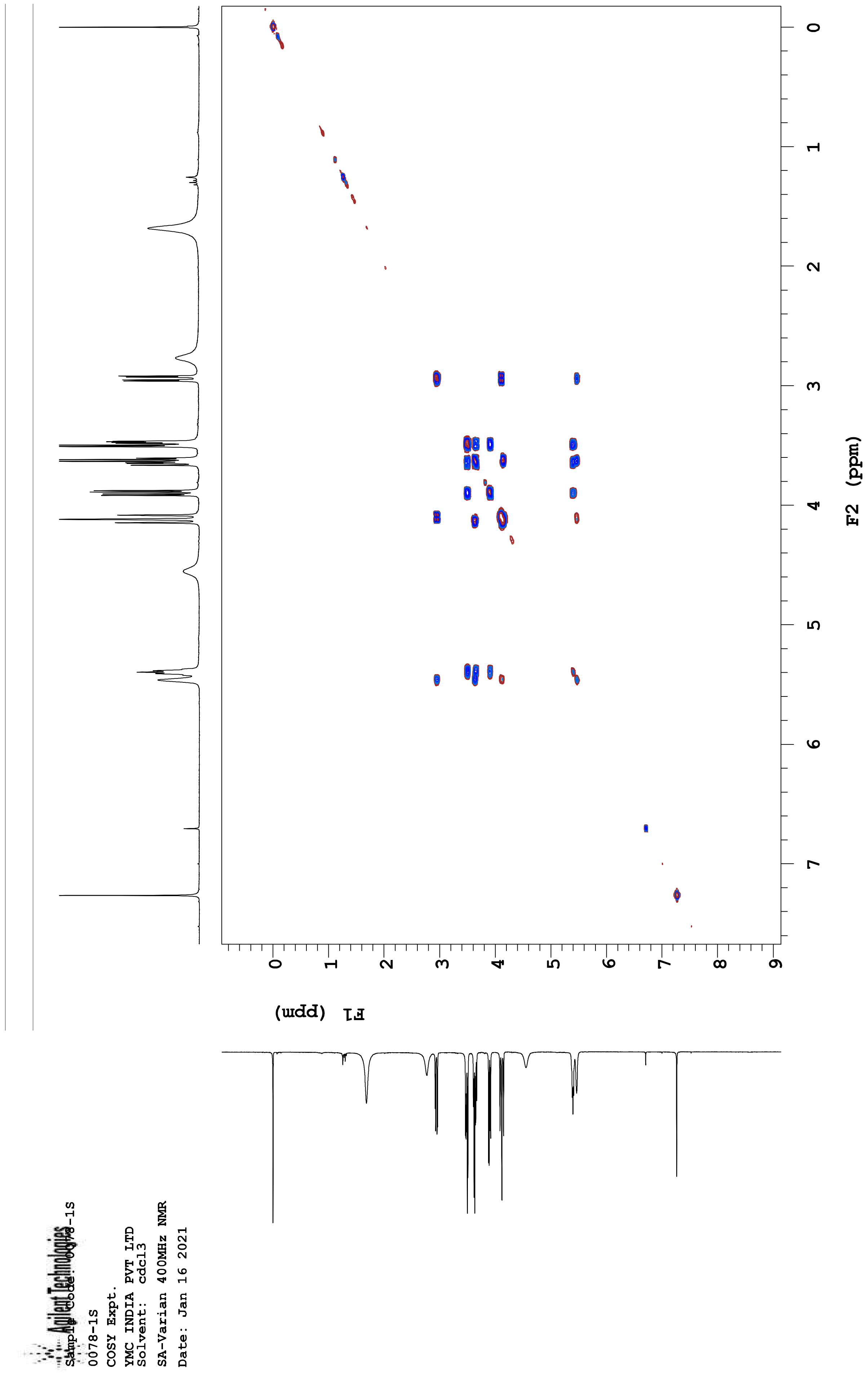
F2 (ppm)
F1 (ppm)
Agilent Technologies
Sample code: 0078-1S
0078-1S
COSY Expt.
YMC INDIA PVT LTD
Solvent: cdcl3
SA-Varian 400MHz NMR
Date: Jan 16 2021

COSY....

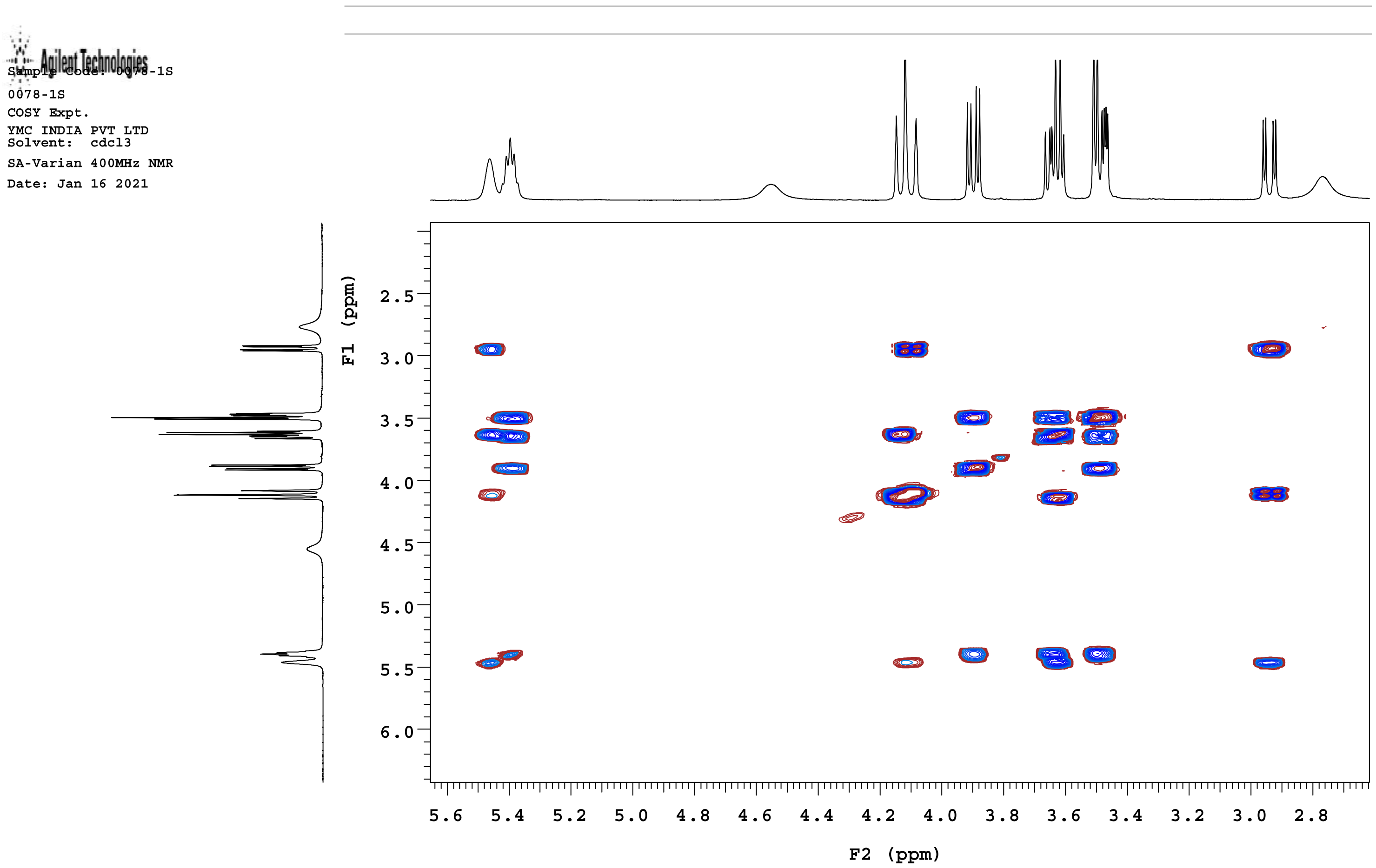
Agilent Technologies
Sample Code: 0078-1S
0078-1S
COSY Expt.
YMC INDIA PVT LTD
Solvent: cdcl3
SA-Varian 400MHz NMR
Date: Jan 16 2021
F1 (ppm)
2.5
3.0
3.5
4.0
4.5
5.0
5.5
6.0
5.6 5.4 5.2 5.0 4.8 4.6 4.4 4.2 4.0 3.8 3.6 3.4 3.2 3.0 2.8
F2 (ppm)

(D) gHSQC

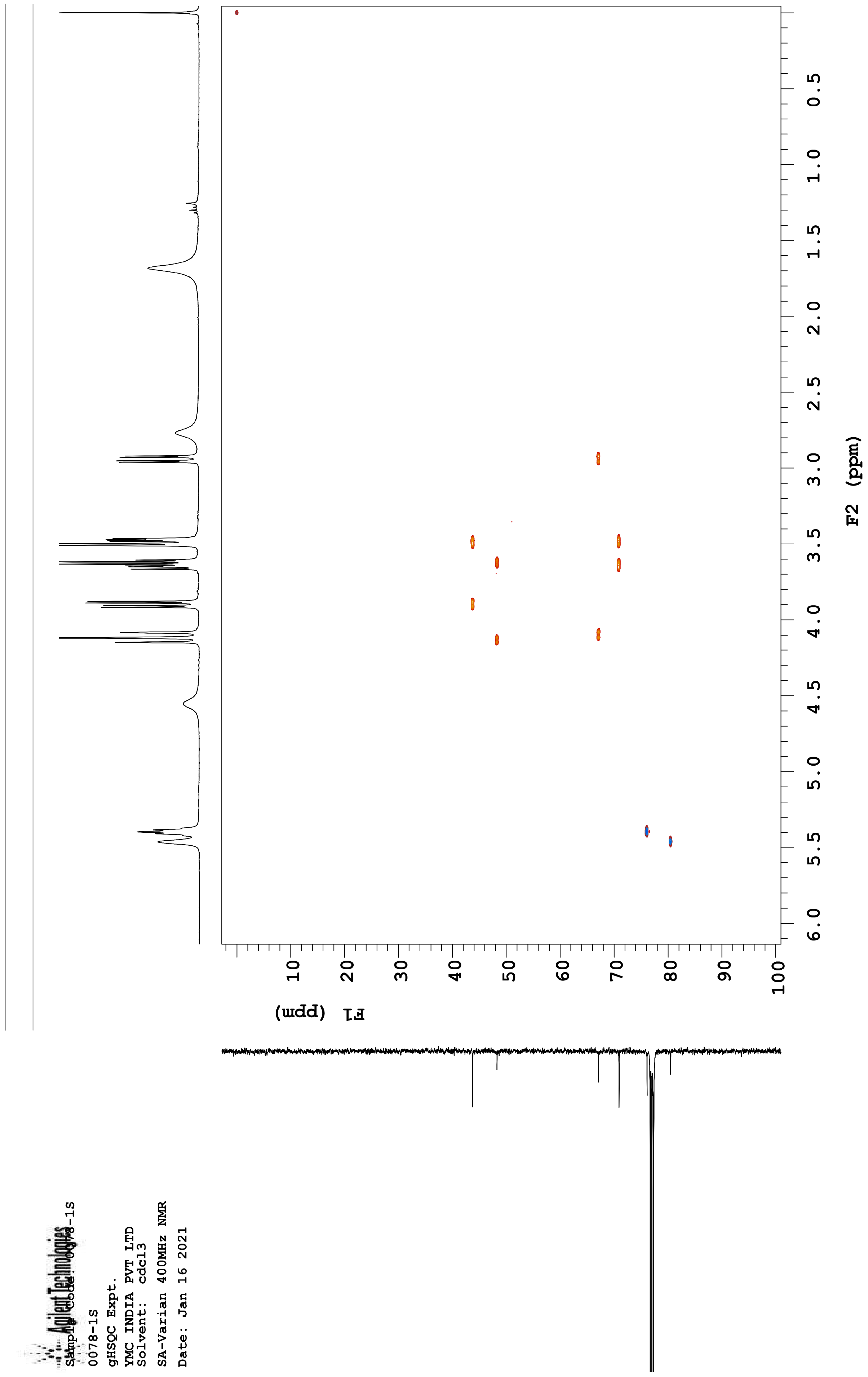
Sample code: 0078-1S
0078-1S
gHSQC Expt.
YMC INDIA PVT LTD
Solvent: cdcl3
SA-Varian 400MHz NMR
Date: Jan 16 2021
F2 (ppm)
F1 (ppm)

gHSQC...

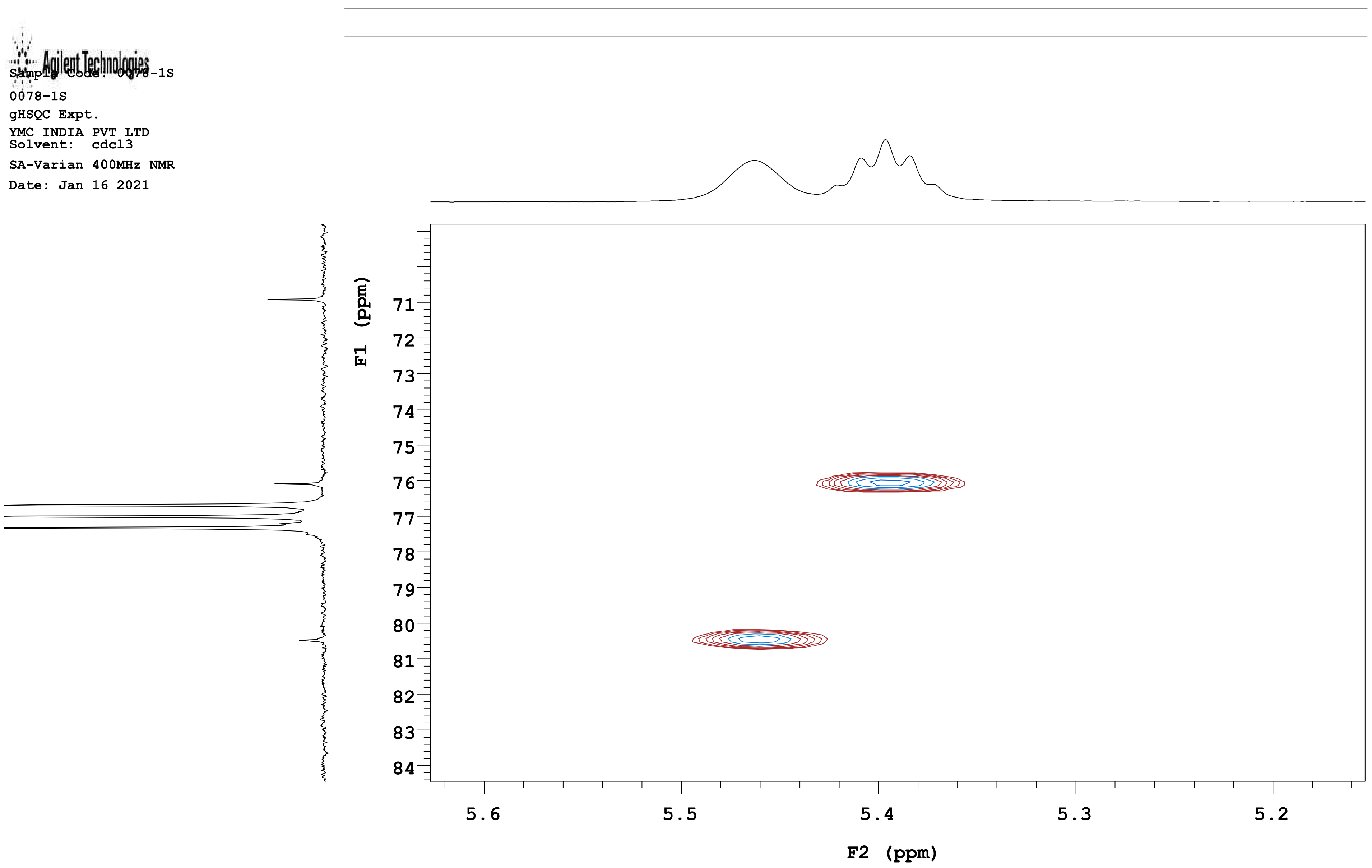
Sample Code: 0078-1S
0078-1S
gHSQC Expt.
YMC INDIA PVT LTD
Solvent: cdcl3
SA-Varian 400MHz NMR
Date: Jan 16 2021
F1 (ppm)
71
72
73
74
75
76
77
78
79
80
81
82
83
84
5.6
5.5
5.4
5.3
5.2
F2 (ppm)

gHSQC....

Sample Code: 0078-1S
0078-1S
gHSQC Expt.
YMC INDIA PVT LTD
Solvent: cdcl3
SA-Varian 400MHz NMR
Date: Jan 16 2021

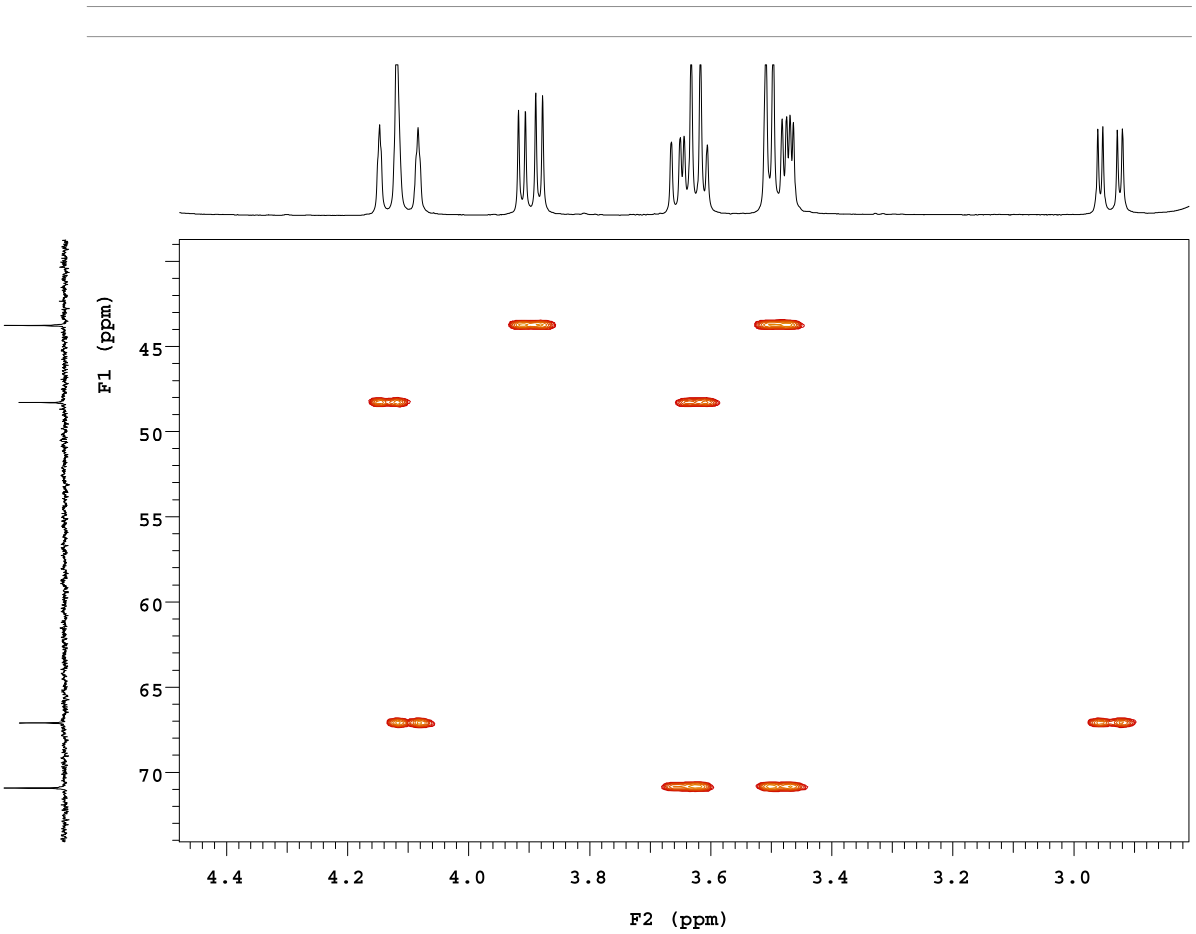

(E) gHMBC

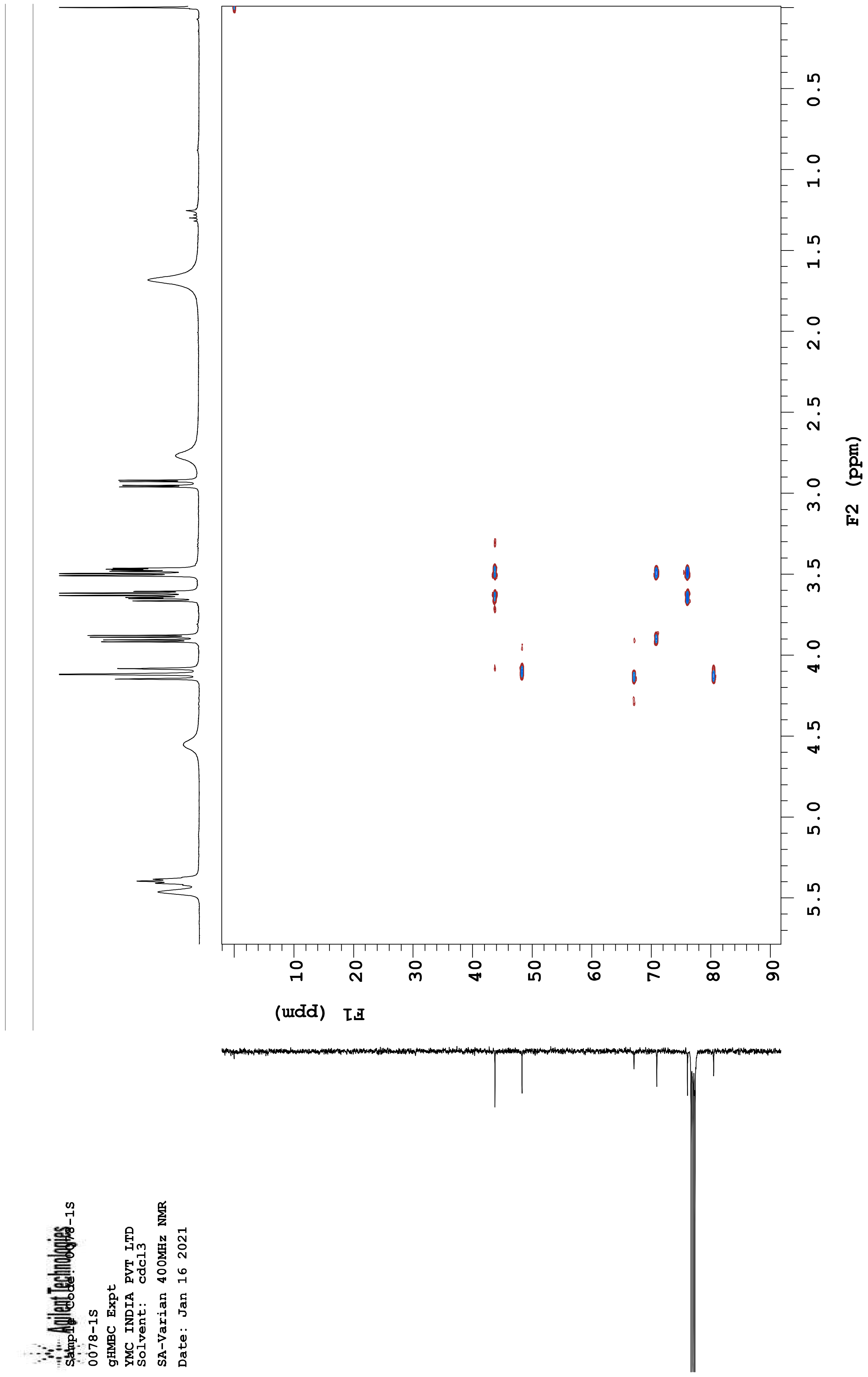
Sample code: 0078-1S
0078-1S
gHMBC Expt
YMC INDIA PVT LTD
Solvent: cdcl3
SA-Varian 400MHz NMR
Date: Jan 16 2021
F2 (ppm)
F1 (ppm)

gHMBC.....

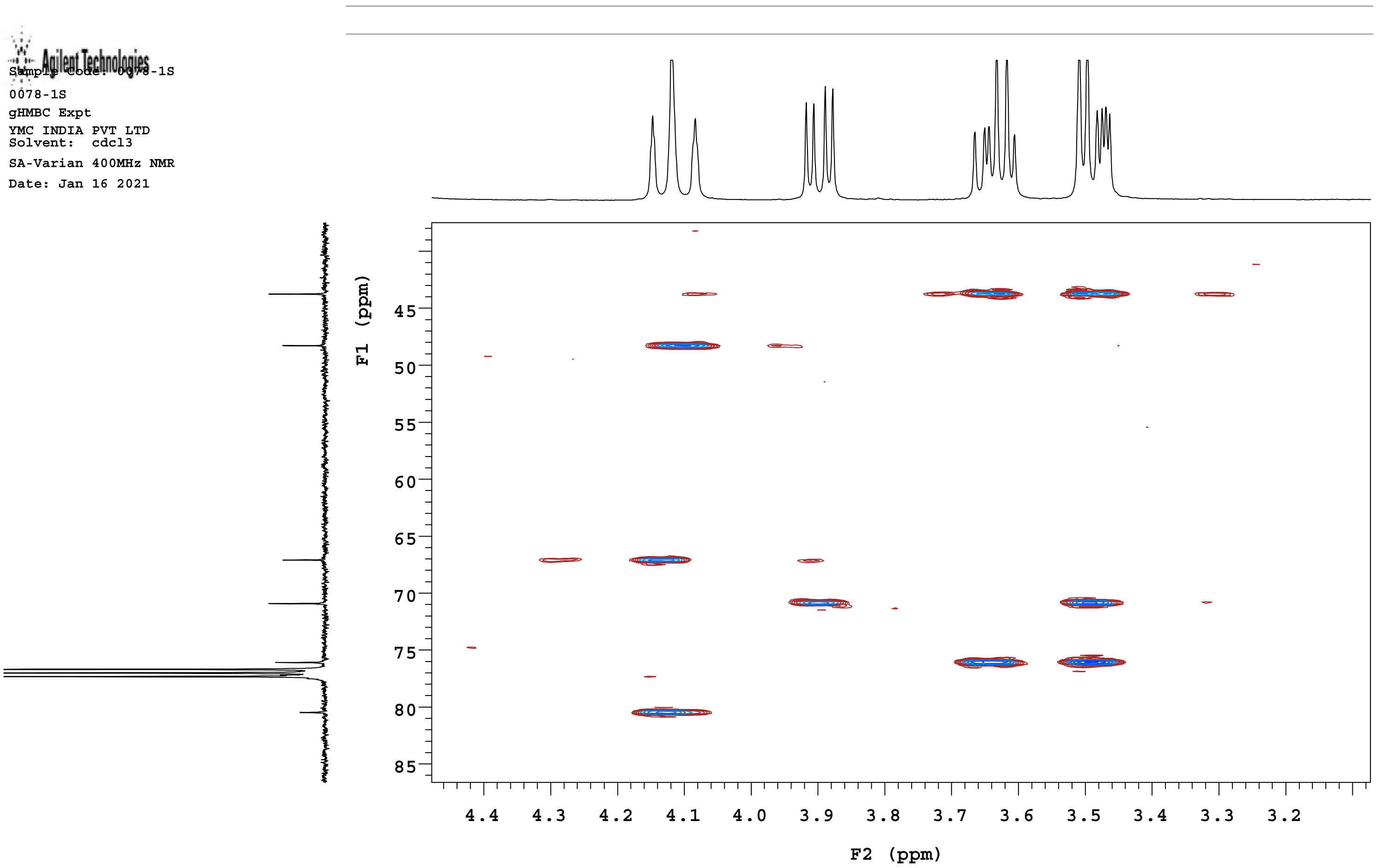

Sample Code: 0078-1S
0078-1S
gHMBC Expt
YMC INDIA PVT LTD
Solvent: cdcl3
SA-Varian 400MHz NMR
Date: Jan 16 2021
F1 (ppm)
45
50
55
60
65
70
75
80
85
4.4
4.3
4.2
4.1
4.0
3.9
3.8
3.7
3.6
3.5
3.4
3.3
3.2
F2 (ppm)

(F) DEPT_45

Sample Code: 0078-1S-DEPT-45

DEPT 45

YMC INDIA PVT LTD

Solvent: CDCl3

SA-Varian 400MHz NMR

Date: Aug 20 2021

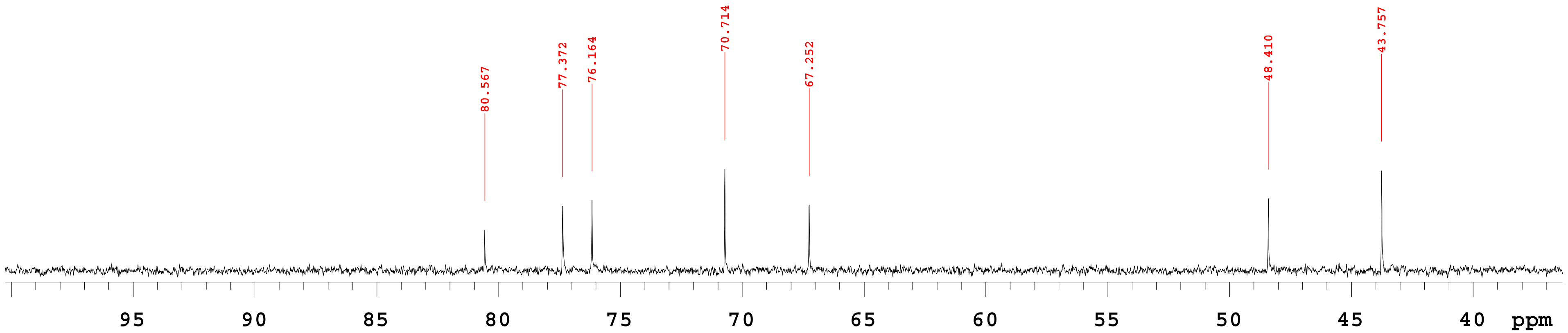

DEPT_90

Sample Code: 0078-1S-DEPT-90

DEPT 90

YMC INDIA PVT LTD

Solvent: CDCl3

SA-Varian 400MHz NMR

Date: Aug 21 2021

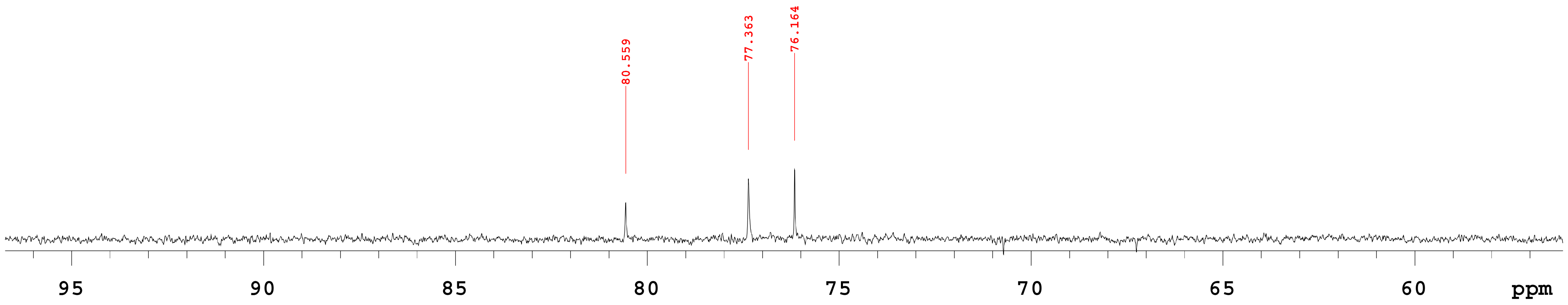

DEPT_135

Sample Code: 0078-1S-DEPT-135

DEPT 135

YMC INDIA PVT LTD

Solvent: CDCl3

SA-Varian 400MHz NMR

Date: Aug 21 2021

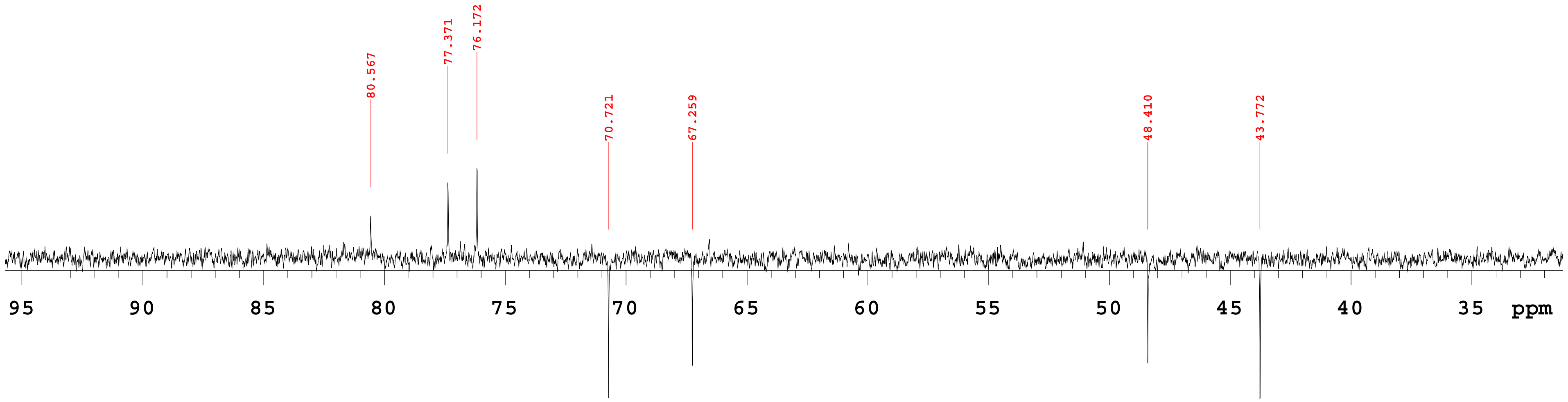

Supplement: Supplementary file 1 [file DataSheet2.PDF]
