# Supplementary material for: A Cyclic Disulfide Diastereomer From Bioactive Fraction of Bruguiera gymnorhiza Shows Anti–Pseudomonas aeruginosa Activity
Source: Front Pharmacol. 2022 Jun 2;13:890790. doi: 10.3389/fphar.2022.890790 (PMC9201687; doi:10.3389/fphar.2022.890790)

Supplementary information 1

Figure S1. Anti-*P. aeruginosa* assay of crude extract and active chromatographic fractions
(A) Well diffusion assay of crude extract with different concentration (10, 20 and 30 mg/mL), (B) column chromatographic fractions 4A and 5A, (C) prep-TLC fraction 4B and 5B (D) HPLC separated fraction BG138 at (A) 32 µg (B) 200 µg of concentration and (E) HPLC chromatogram representing isolation of active fraction 4C, Retention time, 5 min (BG138).

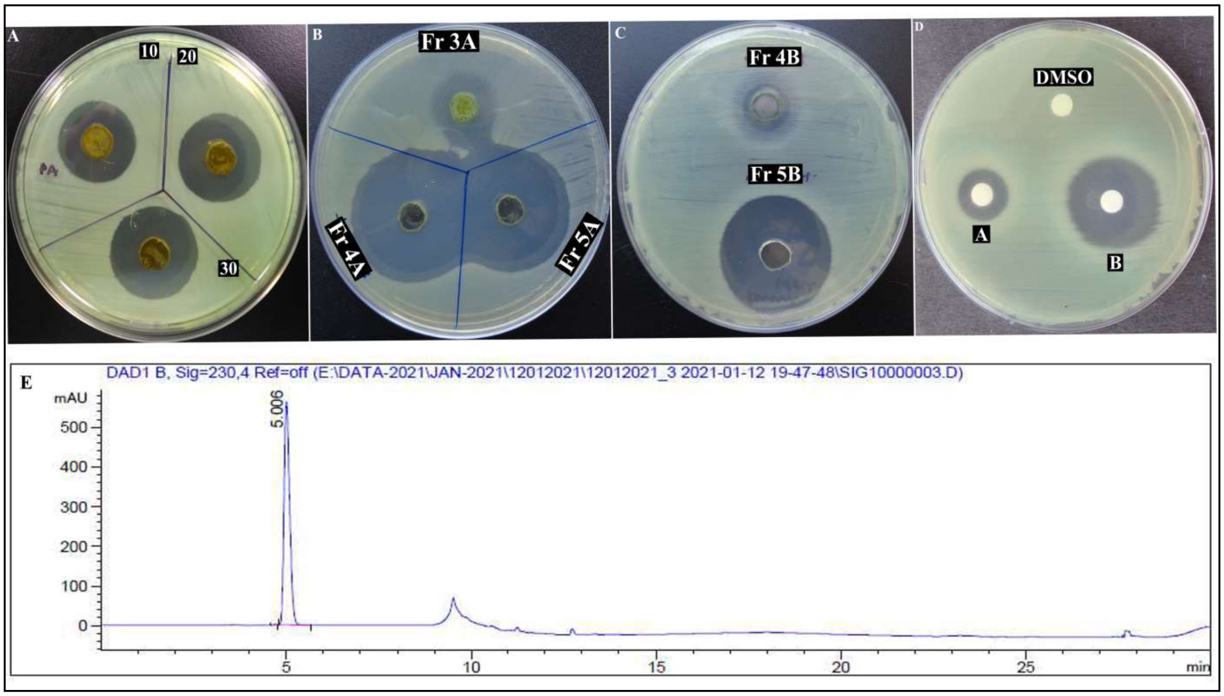

Figure S2. Disc diffusion assay of BG138 against selected bacterial species. (A) *S. aureus*, (B) *S. epidermidis*, (C) *S. pyogenes*, (D) *A. baumannii*, (E) *E. coli*, (F) *K. pneumoniae*, (G) *S. infantis*, (H) *P. putida*, NCIM 2650 (I) *P. syringae* DC3000 and (J) *S. flexneri* MTCC 9543.

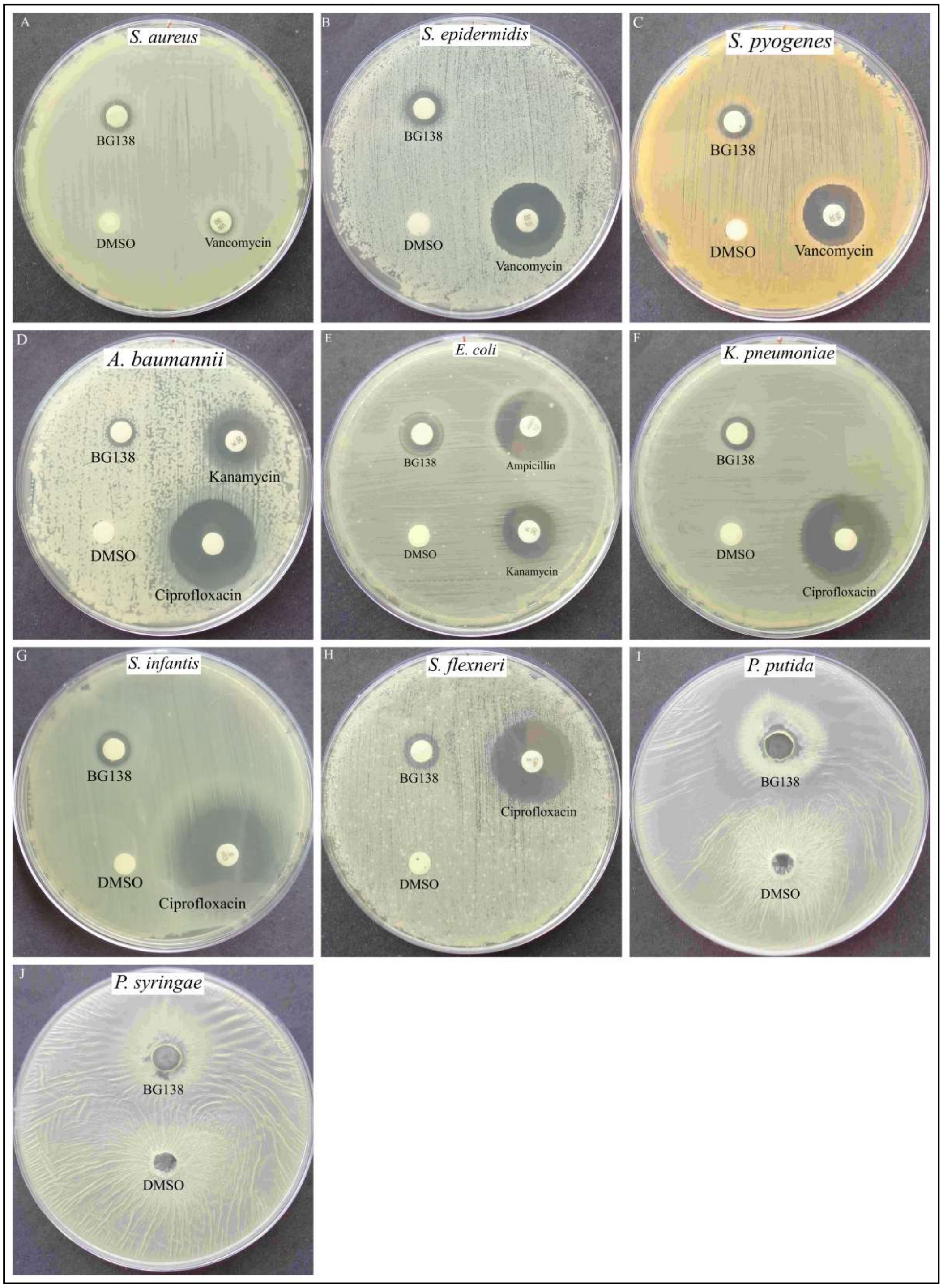

Figure S3. (A) High-resolution mass spectrometry: The molecular mass of the compound detected by HRMS ESI-MS at m/z 138.9870 in positive mode $[M+H]^{+\cdot}$ (B) Fourier transform infrared spectroscopy (FTIR): FTIR spectrum of the BG138.

A)

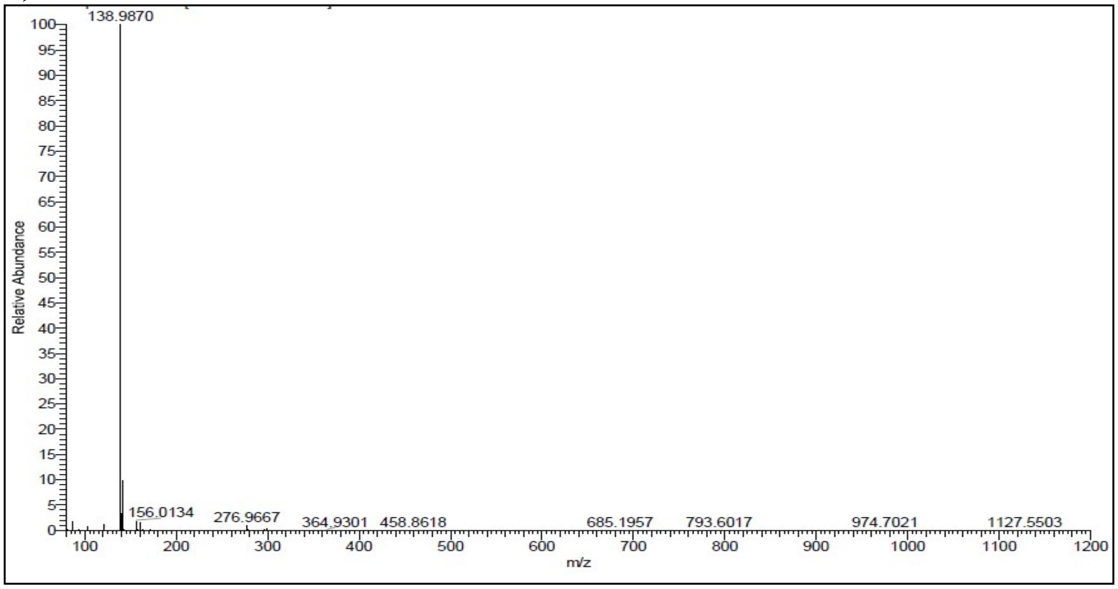

B)

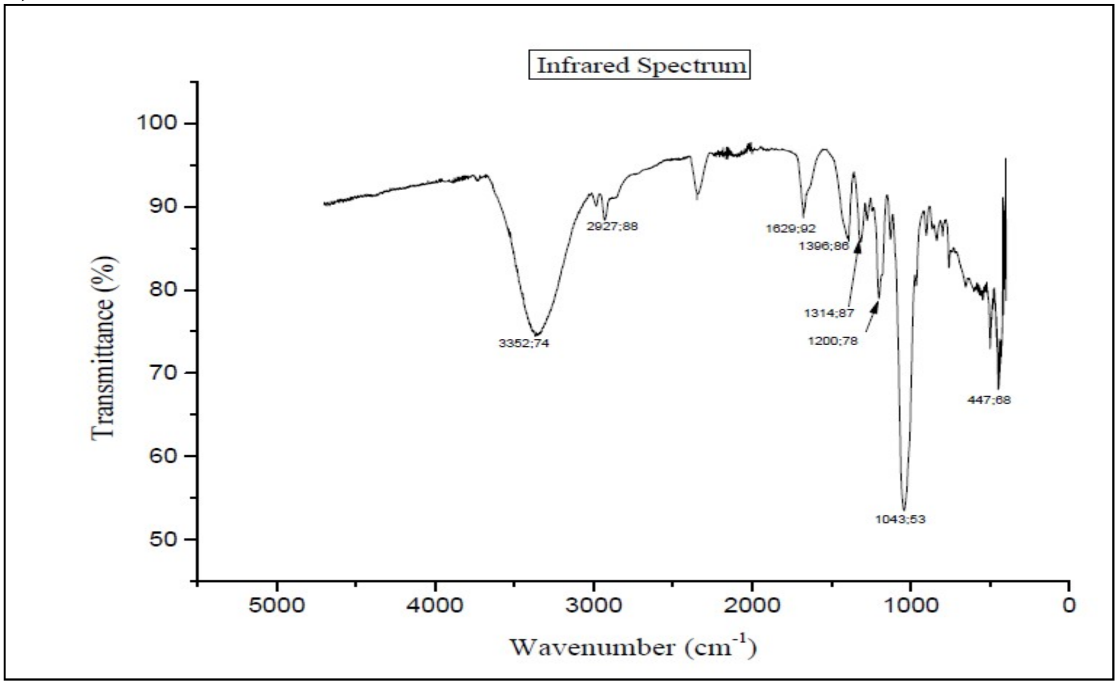

Figure S4. Structure of brugierol and isobrugierol

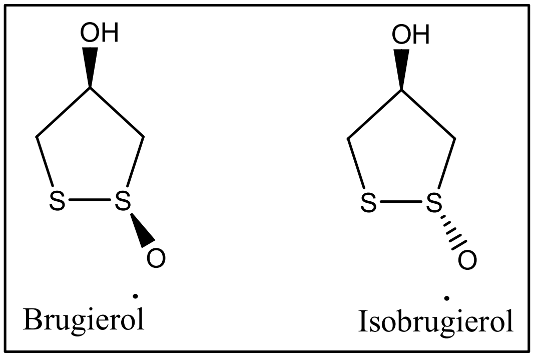

Figure S5. Estimation of rhamnolipids in sterile supernatant after 24 h incubation (A) Estimation without overnight precipitation (B) Estimation after overnight precipitation at 4ºC. Statistical differences were determined by ANOVA followed by Tukey test. $p < 0.05$ (*) $p < 0.001$ (***) versus DMSO control.

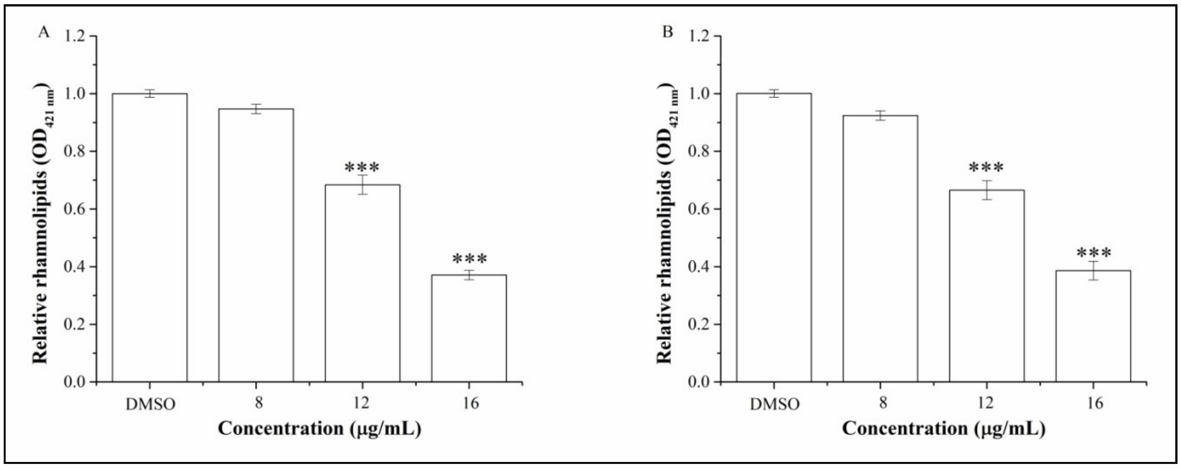

Figure S6. Effect of BG138 on virulence factors secreted by *P. aeruginosa* PA2 after 72 h incubation. Relative levels of virulence factors at different BG138 concentrations (8, 12, and 16 µg/mL) were tested. DMSO was used as negative control. (A) Protease levels, (B) Pyocyanin levels, (C) Pyoverdine levels, (D) Alginate levels, (E) Rhamnolipid levels. Statistical differences were determined by ANOVA followed by Tukey test. $p < 0.05$ (*) versus DMSO control.

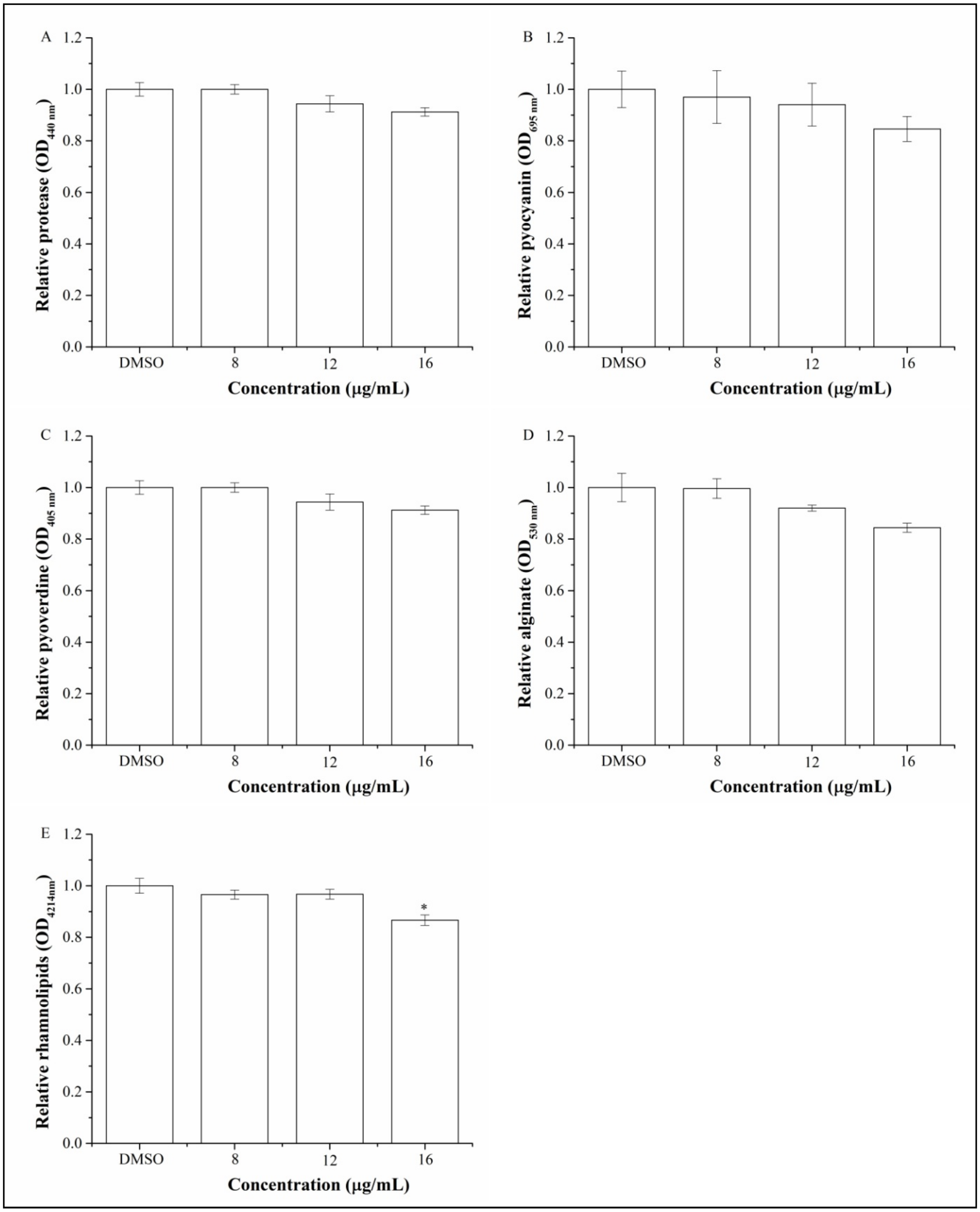

Supplement: Supplementary file 2 [file DataSheet1.pdf]
